# Plasma metabolic disturbances during pregnancy and postpartum in women with depression

**DOI:** 10.1016/j.isci.2022.105666

**Published:** 2022-11-24

**Authors:** Zhiqian Yu, Naomi Matsukawa, Daisuke Saigusa, Ikuko N. Motoike, Chiaki Ono, Yasunobu Okamura, Tomomi Onuma, Yuta Takahashi, Mai Sakai, Hisaaki Kudo, Taku Obara, Keiko Murakami, Matusyuki Shirota, Saya Kikuchi, Natsuko Kobayashi, Yoshie Kikuchi, Junichi Sugawara, Naoko Minegishi, Soichi Ogishima, Kengo Kinoshita, Masayuki Yamamoto, Nobuo Yaegashi, Shinichi Kuriyama, Seizo Koshiba, Hiroaki Tomita

**Affiliations:** 1 Department of Psychiatry, Graduate School of Medicine, Tohoku University, Sendai, Japan; 2 Tohoku Medical Megabank Organization, Tohoku University, Sendai, Japan; 3 Department of Integrative Genomics, Tohoku Medical Megabank Organization, Tohoku University, Sendai, Japan; 4 Laboratory of Biomedical and Analytical Sciences, Faculty of Pharma-Science, Teikyo University; 5 Department of System Bioinformatics, Graduate School of Information Sciences, Tohoku University, Sendai, Japan; 6 Innovations in Next-Generation Medicine, Advanced Research Center, Tohoku University, Sendai, Japan; 7 Division of Disaster Public Health, International Research Institute for Disaster Science, Tohoku University, Sendai, Japan; 8 Department of Disaster Psychiatry, International Research Institute for Disaster Science, Tohoku University, Sendai, Japan; 9 Department of Biobank Life Science, Tohoku Medical Megabank Organization, Tohoku University, Sendai, Japan; 10 Department of Integrative Genomics, Tohoku Medical Megabank Organization, Tohoku University, Sendai, Japan; 11 Department of Medical Biochemistry, Tohoku University Graduate School of Medicine, Sendai, Japan; 12 Department of Gynecology and Obstetrics, Graduate School of Medicine, Tohoku University, Sendai, Japan

**Keywords:** Biological sciences, Physiology, Metabolomics

## Abstract

Examining plasma metabolic profiling during pregnancy and postpartum could help clinicians understand the risk factors for postpartum depression (PPD) development. This analysis targeted paired plasma metabolites in mid-late gestational and 1 month postpartum periods in women with (n = 209) or without (n = 222) PPD. Gas chromatogram-mass spectrometry was used to analyze plasma metabolites at these two time points. Among the 170 objected plasma metabolites, principal component analysis distinguished pregnancy and postpartum metabolites but failed to discriminate women with and without PPD. Compared to women without PPD, those with PPD exhibited 37 metabolites with disparate changes during pregnancy and the 1-month postpartum period and an enriched citrate cycle. Machine learning and multivariate statistical analysis identified two or three compounds that could be potential biomarkers for PPD prediction during pregnancy. Our findings suggest metabolic disturbances in women with depression and may help to elucidate metabolic processes associated with PPD development.

## Highlights

• Maternal plasma metabolite level changes from pregnancy to postpartum
• PPD is related to metabolic disturbances during pregnancy
• Disturbance metabolites enrich the citrate cycle
• Gestational metabolites can predict PPD during pregnancy

## Graphical abstract

**Figure:**
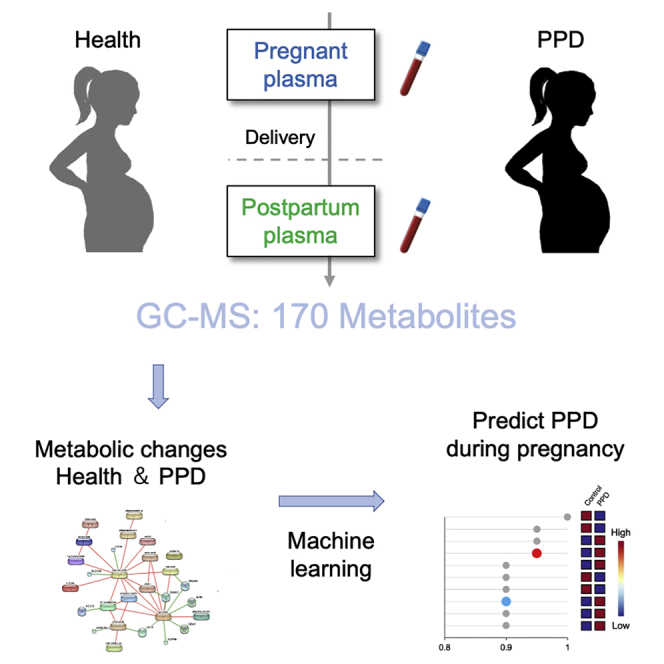

## Introduction

During the postpartum period, 10–20% of women are vulnerable to clinical depression beyond 5 days to 6 weeks after delivery,^1^^,^^2^ which may affect the behavioral and cognitive development of their offspring.^3^^,^^4^^,^^5^ However, the risk factors for postpartum depression (PPD) are not fully understood. Metabolic profiling of postpartum plasma and urine has become a new tool for understanding the pathogenesis of PPD. Metabolic analyses have shown that mothers diagnosed with PPD have increased levels of serum oxidative stress-related metabolites, such as glutathione-disulfide, adenylosuccinate, and ATP, at 8 weeks postpartum.^6^ In addition, urinary metabolites, such as succinate, α-glucose, and dimethylamine, can serve as diagnostic biomarkers for PPD at 8–12 weeks postpartum.^7^ Furthermore, mothers with PPD show alterations in uric metabolites related to amino acid metabolism, neurotransmitter metabolism, and bacterial populations at the sixth week after delivery.^8^ It has been reported that during the peripartum period (3 weeks after delivery), alterations in maternal-origin testosterone and estrogen from the fetal compartment have the strongest correlations with the severity of maternal depressive symptoms.^9^ Moreover, changes in tryptophan levels in maternal plasma, which are decreased in the mid-trimester but return to normal levels after delivery, are known to play a crucial role in pregnancy and the development of PPD.^10^^,^^11^^,^^12^

Importantly, it is well known that normal pregnancy increases plasma volume,^13^ which manifests as a significantly higher plasma volume from the first week of pregnancy, a steepest increase to the maximum volume in the third trimester, and a return to the standard volume after 6 weeks postpartum.^14^^,^^15^ Principal component analysis in another study showed that metabolic profiles change weekly, with a highly choreographed profile, beginning in week 5 of pregnancy, peaking in the postpartum period, and returning to early mid pregnant levels after childbirth.^16^ In general, pregnancy increases plasma and urine-free cortisol and corticosteroid-binding globulin,^17^ and a gas chromatographic analysis revealed that almost all amino acids decrease significantly with increasing gestational period.^18^ Total amino acid concentrations during pregnancy were decreased relative to that at 1 month postpartum, whereas the ratio of essential to non-essential amino acids was increased.^19^ Some metabolic changes during pregnancy influence typical biochemical values, whereas others may be associated with psychiatric disorders like PPD. In women, childbearing years, a period when steroid and peptide hormones can fluctuate dramatically, pose the highest risk of depression.^20^^,^^21^ One study discussed the potential metabolic imprinting mechanism that leads to PPD, whereby pregnant women with depressive symptoms show seasonal differences in glucose and sugar-acid concentrations and the lactate to pyruvate ratio and show abundance of arginine and phosphate.^22^ However, previous studies of women with PPD have included only a limited timeline and number of participants, and the results regarding the metabolic changes of PPD vary greatly.

Considering the pregnancy-induced changes in plasma volume, it is necessary to clarify the different physiological alterations of plasma metabolic profiles in mothers with or without PPD, which can help uncover metabolic risk factors for PPD development. This study aimed to investigate (1) whether plasma metabolic profiling can discriminate between the pregnancy and postpartum periods, (2) whether PPD is associated with plasma metabolite profiles (3) the links between metabolic profiles and the underlying mechanisms of PPD development, and (4) the risk factors and potential metabolic biomarkers for the early prediction of PPD during pregnancy.

## Results

### Demographics characteristics of the cohort

The Japanese version of the Kessler Psychological Distress Scale (K6) and the Japanese version of the Edinburgh Postnatal Depression Scale (EPDS) score were collected at two time points: at 24^th^-27^th^ weeks of gestation and fourth to fifth weeks postpartum. The K6 and EPDS were employed to evaluate mental status during pregnancy and postpartum, respectively, and blood samples were collected at these time points. In Table 1, K6 scores were significantly higher in women with PPD (EPDS ≥9; n = 241) than in the control (EPDS ≤2; n = 250) (p< 0.001). The prevalence of PPD was significantly higher in primiparous women (previous birth = 0) than in multiparous (previous births ≥1) (Pearson’s chi-square test, p< 0.05). Smoking rates during and before pregnancy were highest (p< 0.01) and newborn weight (p = 0.037) and height (p = 0.015) were significantly decreased in women with PPD compared with control. There were no significant differences in blood chemical examination and urine test results between women with and without PPD.

**Table 1:** Demographics characteristics of the participants

Demographics	Control group (EPDS ≦ 2)	PPD group^a^ (EPDS ≧ 9)
Age	(n = 250)	(n = 241)

Mid-late gestational age	30.89 ± 4.95	31.46 ± 3.73

Mental status	(n = 250)	(n = 241)

K6 (Mid-late gestation)	1.28 ± 0.14	7.34 ± 0.34^∗∗∗^
EPDS (One month after delivery)	1.26 ± 0.05	12.50 ± 0.24^∗∗∗^

Body mass index	(n = 223)	(n = 224)

Early gestation (5–13 weeks)	21.48 ± 0.22	21.92 ± 0.24
One month after delivery^b^	22.82 ± 0.23	23.11 ± 0.22

Previous births (%)	(n = 250)	(n = 241)

0	25.68	54.07^∗∗∗^
1	42.33	30.62^∗∗∗^
2	26.13	12.92^∗∗∗^
≧3	5.86	2.39^∗∗∗^

Smoking during pregnancy (%)	(n = 222)	(n = 208)

Yes (during pregnancy)	0.14	5.77^∗∗^
Yes (before pregnancy)	33.33	49.04^∗∗^
Never	65.32	45.19^∗∗∗^

Alcohol during pregnancy (%)	(n = 235)	(n = 240)

Yes (during pregnancy)	20.00	15.74
Yes (before pregnancy)	33.33	34.89
Never	44.58	51.49

Gestational age	(n = 250)	(n = 241)

Gestational weeks of delivery	38.96 ± 0.12	39.06 ± 0.14

**Sex of the newborn**		

Male	114 (46.2%)	141 (55.3%)
Female	133 (53.8%)	114 (44.7%)

Birth weight of newborn (g)	(n = 187)	(n = 213)

Body weight	3043.63 ± 29.60	2935.97 ± 32.33^∗^
Body weight in male^c^	3068.78 ± 48.38	2983.75 ± 44.35
Body weight in female^d^	3020.77 ± 35.68	2877.74 ± 46.72^∗^

Body height of newborn (cm)	(n = 186)	(n = 212)

Body height	49.45 ± 0.16	48.93 ± 0.19^∗^
Body height in male^e^	49.66 ± 0.25	49.19 ± 0.25
Body height in female^f^	49.25 ± 0.19	48.62 ± 0.28

Blood chemical examination^g^	(n = 239)	(n = 241)

GOT (IU/L)	15.58 ± 0.55	15.30 ± 1.13
GPT (IU/L)	10.58 ± 0.61	13.11 ± 2.63
γGTP (IU/L)	9.98 ± 0.46	10.89 ± 0.85
BUN (mg/dL)	8.12 ± 0.22	8.48 ± 0.40
UA (mg/dL)	3.52 ± 0.10	3.33 ± 0.09
Glycoalbumin (%)	13.37 ± 0.13	13.27 ± 0.15
HBA1c (NGSP) (%)	5.00 ± 0.04	5.04 ± 0.05
Triglyceride (mg/dL)	219.76 ± 17.65	227.09 ± 12.63
Total Cholesterol (mg/dL)	265.81 ± 4.72	254.16 ± 4.92
HDL Cholesterol (mg/dL)	77.84 ± 1.77	76.89 ± 1.78
Nonspecific IgE (UA/mL)	216.55 ± 107.20	126.91 ± 33.25
Cystatin C [colloidal gold] (mg/dL)	0.62 ± 0.02	0.59 ± 0.02

Urine tests^g^	(n = 237)	(n = 241)

Chloride (mEq/L)	63.12 ± 9.80	88.28 ± 13.11
Potassium (mEq/L)	21.34 ± 3.77	22.25 ± 3.24

K6: The Japanese version of the Kessler Psychological Distress Scale.EPDS: The Japanese version of the Edinburgh Postnatal Depression Scale.PPD: Postparum depression.BUN: Blood urea nitrogen.UA: Uric acid.NGSP: National Glycohemoglobin Standardization Program.∗ P <0.05, ∗∗ P <0.01, ∗∗∗ P < 0.001 vs. Control group.

a Excluded six women from the PPD group who withdrawn the consent.

b Control group (n=174), PPD group (n=167).

c Birth weight in male (Control group n=90; PPD group n=117).

d Birth weight in female (Control group n=96; PPD group n=96).

e Body height in male (Control group n=90; PPD group n=116).

f Body height in female (Control group n=95; PPD group n=96).

g In the Mid-late gestation.Values are means ± SEM (or percentages).

### Plasma metabolic changes during mid-late gestation and at 1 month postpartum

**Figure 1:**
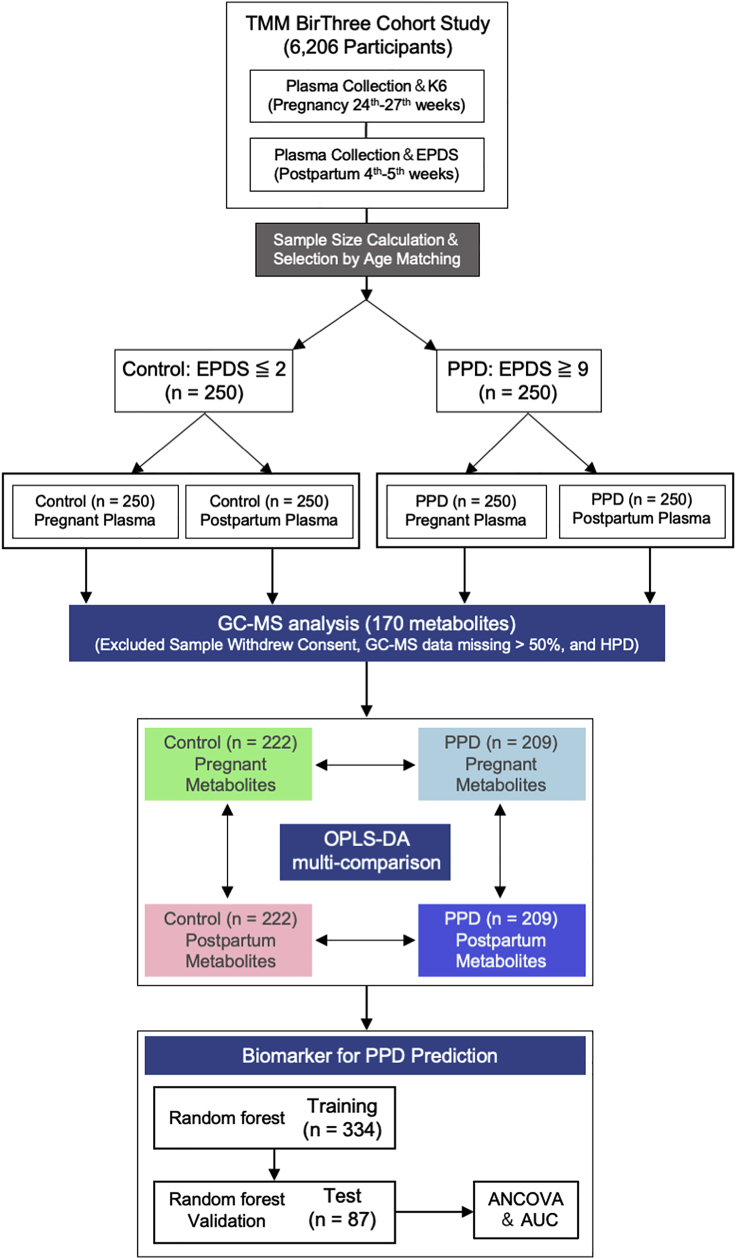
Schema of the study design The study is based on TMM BirThree Cohort Study that included 6,206 mothers with both K6 (24^th^–27^th^ weeks pregnancy) and EPDS scores (1 month postpartum). After calculating the required sample size, 250 women with an EPDS score less than or equal to 2 (Control; EPDS ≤2) and an EPDS score greater than or equal to 9 (PPD; EPDS ≥9) were randomly selected. A total of 500 plasma samples from these women in pregnancy and during postpartum were used for gas chromatogram-mass spectrometry (GC-MS) analyses of 170 metabolites. Excluded those who withdrew consent, with hypertensive pregnancy disorders, and with missing GC-MS data of over 50%, 431 paired samples from the pregnancy and postpartum period (Control, n = 209; PPD, n = 222) were further analyzed using orthogonal projections to latent structures discriminant analysis (OPLS-DA) and multi-comparison. Potential metabolic biomarkers for PPD prediction during pregnancy were assessed and validated in training (n = 334) and test (n = 87) datasets using machine learning (Multivariate ROC curve based exploratory analysis based on random forest). The expression and prediction power of candidate biomarkers during pregnancy were statistically evaluated by ANCOVA and receiver operating characteristic (ROC) curve with covariates including age, BMI, smoking, and K6 scores. K6: The Japanese version of the Kessler Psychological Distress Scale. EPDS: The Japanese version of the Edinburgh Postnatal Depression Scale.

We investigated whether the expression of plasma metabolites was associated with PPD during pregnancy and postpartum periods and whether they were potential biomarkers for PPD prediction (Figure 1). We paired the participants to prevent interindividual variations in metabolic changes. We determined whether changes in plasma metabolites occur from mid-late gestation to 1 month postpartum. There were no significant differences in the baseline characteristics between participants with PPD (n = 209) and without PPD (n = 222) (age: p = 0.06; BMI: p = 0.17) after we removed the unpaired subjects. Among the paired participants in the group with EPDS scores ≥9, the range of EPDS scores was 9–30, and the numbers of participants in each score category were strongly correlated with the distribution of the ToMMo cohort (R = 0.983; p< 0.0001) (Figure S1A in the supplemental information). A total of 170 metabolites, automatically confirmed in the gas chromatogram-mass spectrometry (GC-MS) database in 431 available paired gestational and postpartum maternal plasma samples (controls: n = 222 and PPD: n = 209), were normalized and used for the analyses (Figure S1B in supplemental information). Orthogonal projections to latent structures discriminant analysis (OPLS-DA; R^2^Y cum = 0.924, *Q*^2^cum = 0.895) divided the metabolites into two discriminated groups with few overlaps, indicating the distinct changes in metabolite profiles from mid-late gestation to 1 month postpartum.

**Figure 2:**
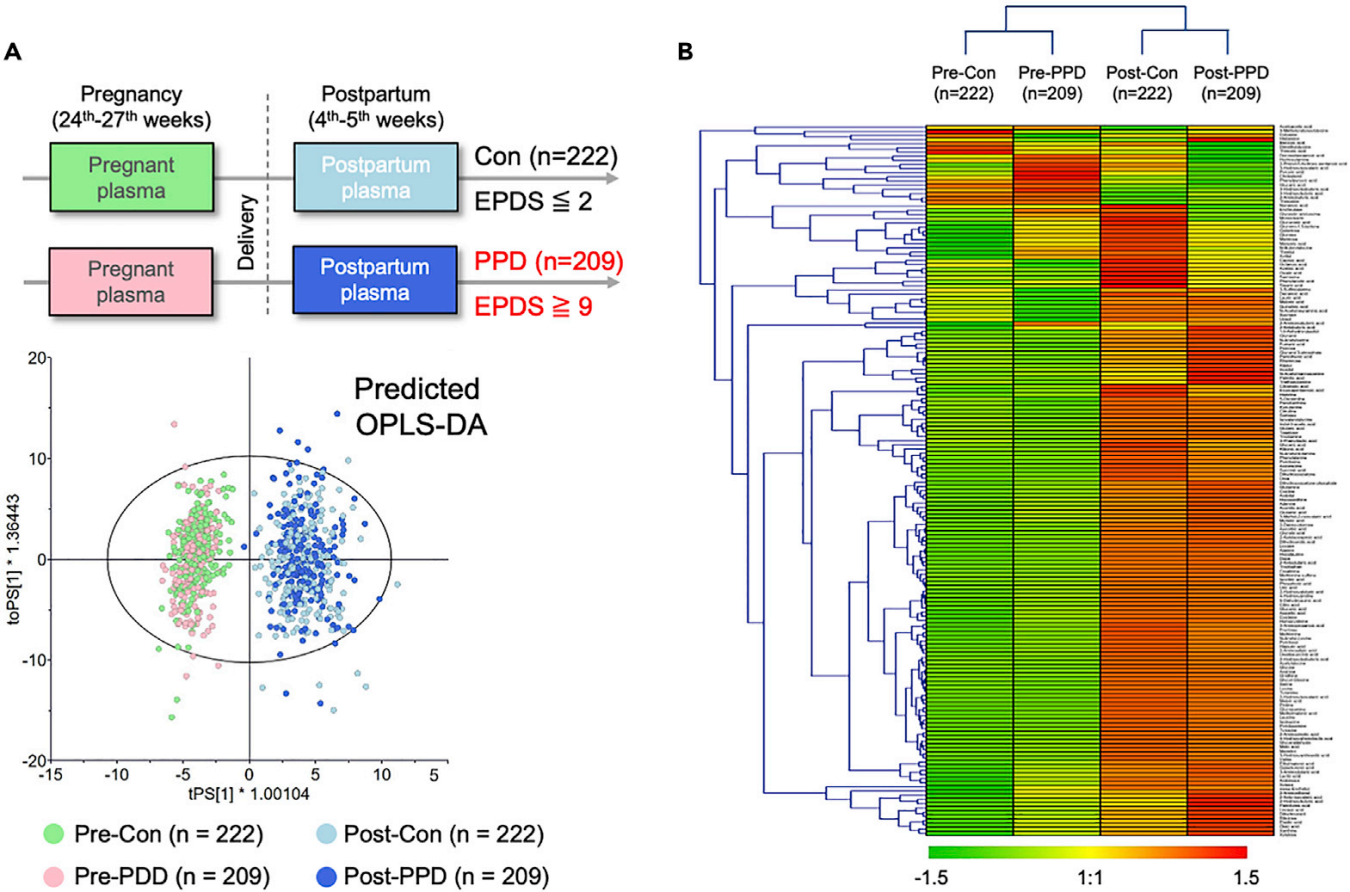
Pregnant and postpartum plasma metabolic profiling between women with and without postpartum depression (A) Predicted OPLS-DA score plots of each woman’s paired plasma metabolites at pregnancy and postpartum in those without postpartum depression (PPD) (controls: EPDS ≤2; n = 222) or with PPD (EPDS ≥9; n = 209). (B) Heatmap and clustering analysis visualizing metabolic differentiation of metabolites during pregnancy and at postpartum. The average signal intensity of the prenatal, and postpartum metabolites of each woman without PPD (EPDS ≤2; n = 222) or with PPD (EPDS ≥9; n = 209) are reported. OPLS-DA, Orthogonal Projections to Latent Structures Discriminant Analysis; EPDS, the Japanese version of the Edinburgh Postnatal Depression Scale; PPD, postpartum depression; Pre-Con, plasma metabolites in pregnancy in controls; Pre-PPD, plasma metabolites in pregnancy in women without PPD; Post-Con, plasma metabolites postpartum in controls; Post-PPD, plasma metabolites postpartum in women with PPD. EPDS: The Japanese version of the Edinburgh Postnatal Depression Scale.

Next, we confirmed the effects of PPD on metabolite levels. Gestational and postpartum maternal plasma samples from paired control and PPD groups, were separated into four groups (Figure 2B). OPLS-DA divided the four groups into two major discriminating groups (Figure 2A) including the pregnant control (green plots, n = 222) and pregnant PPD (pink plots, n = 209) groups and the postpartum control (light blue plots, n = 222) and postpartum PPD (dark blue plots, n = 209) groups. These results indicate a significant impact of “delivery” from pregnancy to postpartum but a limitation of “PPD” regarding metabolite changes. Further hierarchical clustering (Figure 2B, See also Table S1) showed extensive metabolic changes in postpartum plasma compared with pregnancy plasma in the control and PPD groups. In Figure 2B, several unique patterns of metabolite changes in both pregnant and postpartum states for the control and PPD groups were observed, which may be affected by prenatal mental distress or PPD.

### Effects of depression on plasma metabolic changes

**Figure 3:**
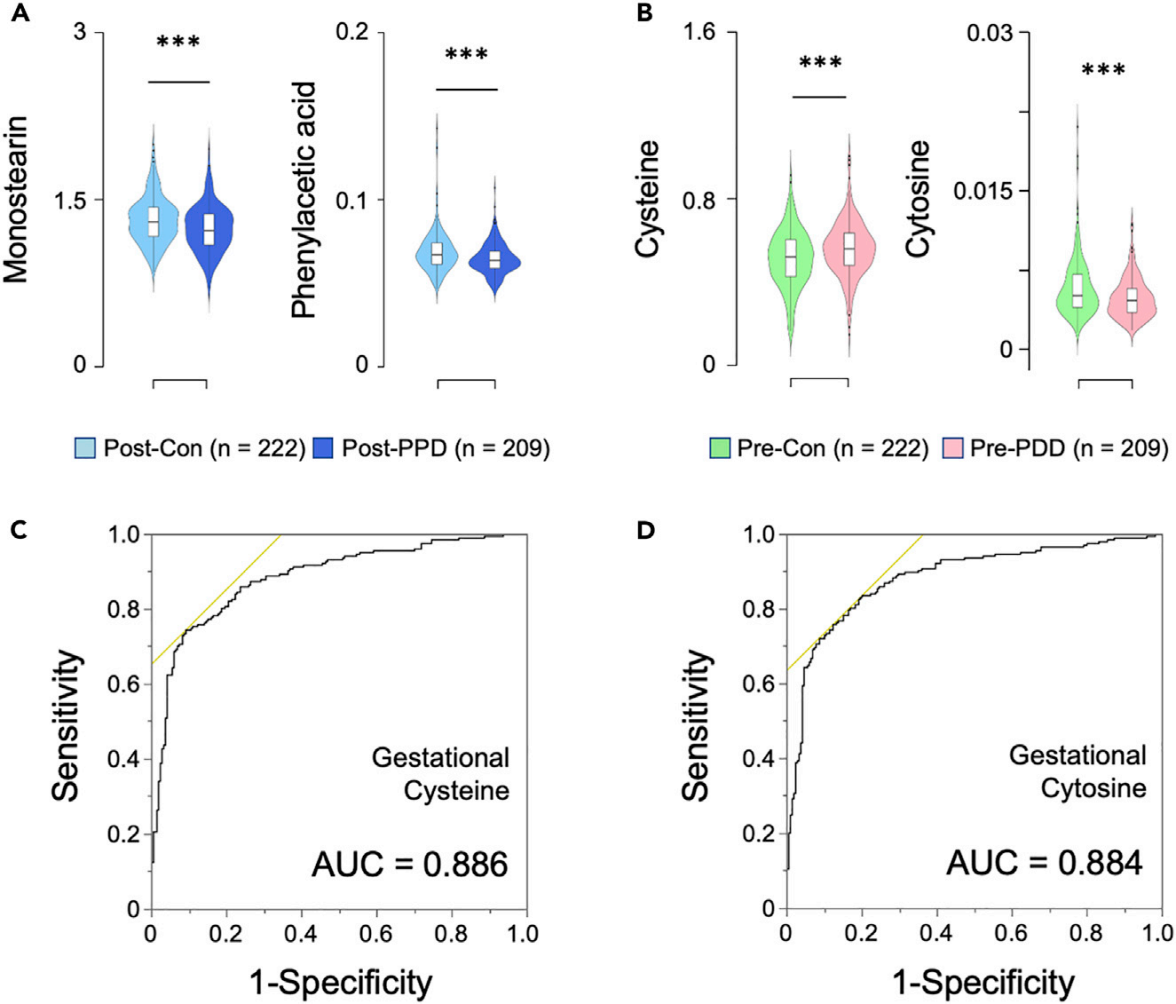
Significantly changed plasma metabolites in women with postpartum depression (A) Violin plots of plasma monostearin and phenylacetic acid levels at the postpartum period in the control (EPDS ≤2; n = 221) and PPD (EPDS ≥9; n = 207) groups (FDR q-value <0.05). Violin plots show the frequency (density plot width) of each group with 95% confidence intervals, interquartile ranges, and median values. Post-PPD versus Post-Con, p< 0.001. Statistical significance measured by paired Student’s *t* test with false discovery rate (FDR) correction. (B) Violin plots of plasma cysteine and cytosine levels during the pregnancy period in the control (EPDS ≤2; n = 221) and PPD (EPDS ≥9; n = 207) groups (FDR q-value <0.05). Pre-PPD versus Pre-Con, p< 0.001. Statistical significance measured by paired Student’s *t* test with FDR correction. (C) Receiver operating characteristic (ROC) analysis of EPSD scores and cysteine levels during the pregnancy period based on the EPDS cutoff score of 2/9. (D) ROC analysis of EPSD scores and cytosine levels during the pregnancy period based on the EPDS cutoff score of 2/9. EPDS, the score of the Japanese version of the Edinburgh Postnatal Depression Scale; Pre, metabolites at mid-late pregnancy; Post, metabolites at 1 month postpartum;-Con, control;-PPD, women with PPD. ^∗∗∗^p< 0.001. K6: The Japanese version of the Kessler Psychological Distress Scale.

We examined postpartum plasma metabolic differences between the PPD (n = 209) and control (n = 222) groups. OPLS-DA (R^2^Y cum = 0.114, *Q*^2^cum = −0.005) was unable to discriminate postpartum metabolites between the groups. After FDR multiple comparison correction, only two metabolites, monostearin and phenylacetic acid, were significantly decreased in the plasma of the PPD group compared with the controls (Figure 3A; FDR q-value <0.05; See also Table S2). Furthermore, we examined plasma in pregnancy to confirm the initially altered metabolites that potentially contribute to PPD development, though OPLS-DA was unable to discriminate gestational metabolites by PPD status (R^2^Y cum = 0.094, *Q*^2^cum = 0.007). Nevertheless, FDR correction revealed two gestational metabolites, namely, significantly increased cysteine and decreased cytosine in the PPD group compared with controls (Figure 3B; FDR q-value <0.05; See also Table S3). The area under the curve (AUC), odds ratio (OR) and 95% confidence interval (CI) of the receiver operating characteristic curve (ROC) curves were calculated to examine clinical risk prediction ability of the two gestational biomarkers to discriminate women with or without PPD by EPDS score. After adjusting for BMI, age, smoking, and K6 scores, our results suggests that significantly higher levels of cysteine (AUC = 0.886, OR = 8.444, 95% CI 1.719–43.698; Figure 3C) and lower levels of cytosine (AUC = 0.884, OR = 0.858, 95% CI 0.752–0.968; Figure 3D) may be a molecular risk factor for PDD prediction during mid–late pregnancy. The K6 scores were significantly higher in women with PPD compared with those without PPD. After excluding the K6 scores from the covariates, the discriminative values of AUC were higher than 0.6 in cysteine (AUC = 0.661, OR = 9.549, 95% CI 2.674–35.796) and cytosine (AUC = 0.669, OR = 0.816, 95% CI 0.730–0.904).

### Changes in the plasma metabolic profile from pregnancy to postpartum

We determined whether gestational metabolite levels were possibly affected by psychological distress. The metabolites identified in 431 pregnant participants were separated into the following four groups based on simultaneously collected K6 scores: control women (K6 ≤4; n = 281) and women who exhibited low (5 ≤ K6 ≤ 9; n = 82), moderate (10 ≤ K6 ≤ 12; n = 24), and high (13 ≤ K6 ≤ 24; n = 44) levels of psychological distress (see STAR methods). OPLS-DA was unable to discriminate metabolic patterns for each group (Figure S2A in supplemental information). Given the slight differences among the groups that did not achieve statistical significance (FDR q value >0.05), gestational metabolites were apparently not affected by psychological distress during pregnancy (Figure S2B, See also Table S4).

**Figure 4:**
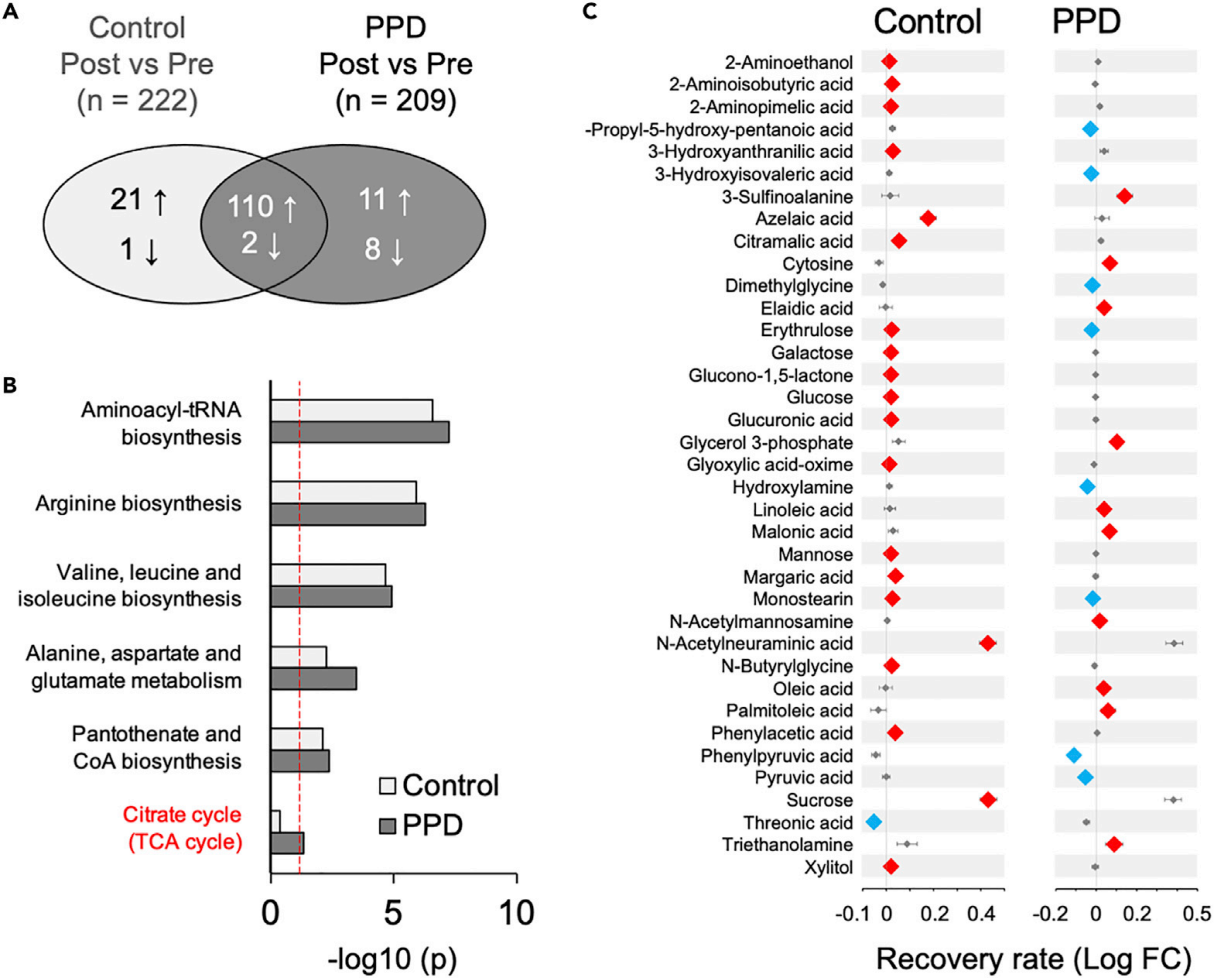
Different metabolic changes and biological networks (A) Venn diagram summarizing the number of metabolites that changed from mid-late gestation to 1 month postpartum in the control and postpartum depression (PPD) groups and the metabolite changes that overlapped (FDR q-value <0.05). (B) Metabolic pathways with significantly changed metabolites from mid-late gestation to 1 month postpartum in control and PPD groups (p< 0.05 with Holm–Bonferroni correction). Redword denotes the different pathway between control and PPD groups. Red dotted line indicates the value of-Log (0.05). (C) Forest plots showing fold changes (FC) (postpartum metabolite signal/pregnant metabolite signal) of different significantly changed 37 metabolites from mid-late pregnancy to 1 month postpartum in control (left) and PPD (right) group, respectively (FDR q-value <0.05). Log FC values are shown in mean ± SEM.

Paired pregnant and postpartum plasma from the control and PPD groups were analyzed, respectively, by paired Student’s *t*-test with FDR correction. In the control group, multiple comparisons revealed 131 significantly increased metabolites and 3 significantly decreased metabolites in postpartum plasma compared with pregnancy plasma (Figure 4A; FDR q-value <0.05; See also Table S5). Furthermore, multiple-comparison paired Student’s *t* test with FDR-correction; analyses revealed 121 significantly increased and 10 significantly decreased metabolites in postpartum plasma compared with pregnancy plasma in the PPD group (Figure 4A; FDR q-value <0.05; See also Table S6). We hypothesized that metabolic network is potentially associated with PPD development. Metabolite set enrichment analysis (MSEA) revealed that the metabolites that changed from pregnancy to postpartum were significantly enriched in 5 metabolic pathways (Holm-Bonferroni adjusted P-value <0.05; Figure 4B, See also Table S7). In addition, metabolites that changed significantly in the PPD group from pregnancy to the postpartum period were significantly enriched in six metabolic pathways (Holm adjust P-value <0.05; Figure 4B, See also Table S8). The “Citrate cycle” (TCA cycle) did not reach statistical significance in the control group, but it changed significantly in the PPD group as well as five other metabolic pathways, “Aminoacyl-tRNA biosynthesis,” “Arginine biosynthesis,” “Valine, leucine, and isoleucine biosynthesis,” “Alanine, aspartate, and glutamate metabolism,” and “Pantothenate and CoA biosynthesis.”

Compared with metabolites that changed significantly from pregnancy to postpartum in the control group, 37 metabolites showed different alterations in the PPD group (Figure 4C, See also Table S9). Among 37 non-overlapping metabolites, two metabolites, erythrulose and monostearin showed opposite changes between the control and PPD groups. The protein-metabolite network was built in the STITCH database^23^ (Figure S3 in the supplemental information). Furthermore, we constructed a regularized partial correlation network based on the Pearson correlation relationship, and different metabolic recovery rates between control and PPD groups that were significantly enhanced or reduced tended to cluster together between the control and PPD groups (Figure S4 in the supplemental information). These findings highlight that although the recovery rate of each metabolite differed between the control and PPD groups, the highly coordinated metabolite regulatory network suggests that a programmed change in plasma metabolism at the system level underlies PPD development.

### Identification of potential gestational metabolic biomarkers for PPD prediction by machine learning

**Figure 5:**
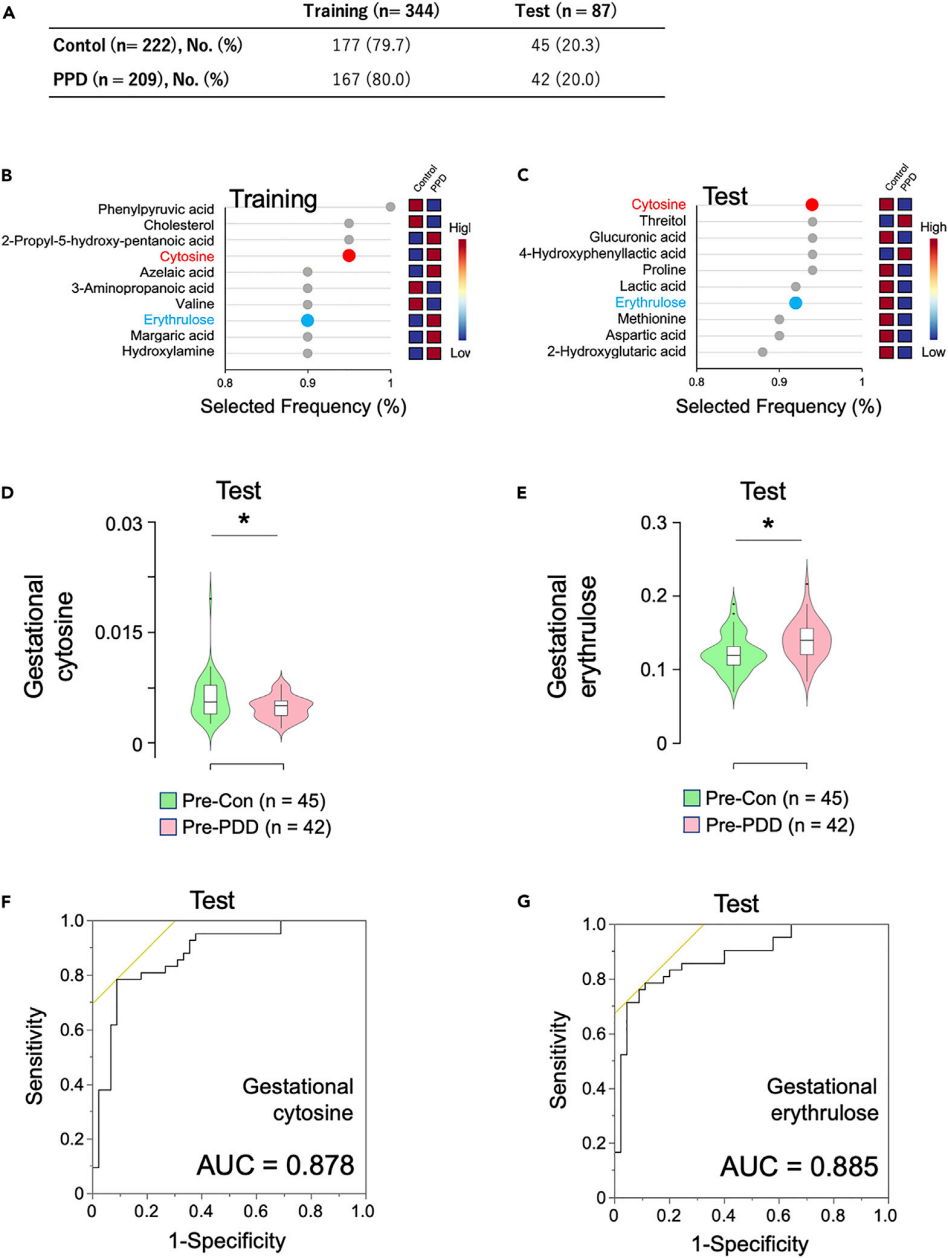
Predictive risk factors selected by machine learning and multi-comparisons (A) The method of hold-out validation was used to evaluate risk metabolites of postpartum depression (PPD) during pregnancy. A total of 431 participants were randomly assigned as 80% for the training and 20% for the test datasets. (B and C) ROC curves are generated by Monte-Carlo cross validation (MCCV) using balanced sub-sampling in each of training and test dataset: two-thirds (⅔) of the samples are used to evaluate the feature importance. The top important features are then used to build classification models which is validated on the ⅓ the samples that were left out based on random forest. The procedure was repeated 10 times to calculate the performance and confidence interval of each model. The top 10 metabolites for PPD prediction, which ranked by plot as percentage of selected frequency from the most important features in training (B) and test (C) datasets. Red circle, Cytosine. Blue circle, Erythrulose. (D) Violin plots of plasma cytosine levels at pregnancy period of women with (n = 45) and without PPD (n = 42). Violin plots show the frequency (density plot width) of each group with 95% confidence intervals, interquartile ranges, and median values. Pre-PPD versus Pre-Con p < 0.05. Statistical significance measured by ANCOVA with covariates including age, BMI, smoking, and K6 scores. (E) Violin plots of plasma erythrulose levels at pregnancy period of women with (n = 45) and without PPD (n = 42). Pre-PPD versus Pre-Con p < 0.05. Statistical significance measured by ANCOVA with covariates including age, BMI, smoking, and K6 scores. (F) ROC analysis of cytosine levels in the test dataset at pregnancy period based on the EPDS cutoff score of 2/9. (G) ROC analysis of erythrulose levels in the test dataset at pregnancy period based on the EPDS cutoff score of 2/9. EPDS, the score of the Japanese version of the Edinburgh Postnatal Depression Scale. PPD, postpartum depression; Pre-Con, plasma metabolites in pregnancy in controls; Pre-PPD, plasma metabolites in pregnancy in women without PPD. ^∗^p< 0.05. K6: The Japanese version of the Kessler Psychological Distress Scale. EPDS: The Japanese version of the Edinburgh Postnatal Depression Scale.

We used the hold-out validation method to avoid the overlap between training and test data.^24^ Based on cohort ID, the recovery rate of each metabolite (postpartum metabolite signal/gestational metabolite signal) in the control and PPD groups were randomly separated as 80% for training (control: n = 177 and PPD: n = 167) and 20% for test (control: n = 45 and PPD: n = 42) datasets (Figure 5A). The recovery rates were combined into the biomarkers panel by discrimination between the control and PPD groups in the MetaboAnalyst.^25^ ROC curves based on the 10-fold cross-validation performance with random forest measured the importance plot of the biomarkers in training and in the test datasets. In the training dataset, the top 10 metabolites that significantly contributed to the discrimination between the control and PPD groups are in the following order: phenylpyruvic acid > cholesterol > 2-propyl-5-hydroxy-pentanoic acid > cytosine > azelaic acid > 3-aminopropanoic acid > valine > erythrulose > margaric acid > hydroxylamine (Figure 5B, See also Table S10). Validated analysis in the test dataset showed that the top 10 significant metabolites were in the following order: cytosine > threitol > glucuronic acid > 4-hydroxyphenyllactic acid > proline > lactic acid > erythrulose > methionine > aspartic acid > 2-hydroxyglutaric acid (Figure 5C, See also Table S11). Two metabolites, cytosine and erythrulose, that were commonly represented in the training and test datasets were further evaluated.

The gestational cytosine and erythrulose levels in the PPD were further compared with the control. After adjusting for BMI, age, smoking, and K6 score, significantly lower levels of gestational cytosine (Figure 5D; F_5, 86_ = 2.607, p = 0.031) and higher levers of gestational erythrulose (Figure 5E; F_5, 86_ = 3.171, p = 0.012) were confirmed in the PPD. These results were consistent with the comparison in the training data (Figures S5A and S5B in the supplemental information). Furthermore, AUC, OR, and 95% CI of the ROC curve were calculated to determine the possibility of gestational cytosine and erythrulose to discriminate between the control and PPD groups based on the EPDS score. In the test dataset, gestational cytosine and erythrulose had high AUC values of 0.878 (OR = 0.699, 95% CI 0.482–0.981; Figure 5F) and 0.885 (OR = 146.128, 95% CI 4.506–1084.86; Figure 5G) in discriminating PPD status may be considered as predictors during pregnancy. When the K6 score, which was significantly higher in the PPD than in the control group was excluded from the covariates, the discriminative values were higher than 0.6 for cytosine (AUC = 0.697, OR = 0.665, 95% CI 0.479–0.881) and for erythrulose (AUC = 0.718, OR = 110.652, 95% CI 6.868–291.488). In addition, gestational cytosine (AUC = 0.887) and erythrulose (AUC = 0.889) of the training dataset also had high AUC values in discriminating PPD status (Figures S5C and S5D in the supplemental information).

## Discussion

This study perform the profiling of metabolic changes from the mid-late gestational period to 1-month postpartum in women with and without PPD. Normal pregnancy increases plasma volume, which manifests as a significantly higher plasma volume from the first week of pregnancy, a steepest increase to a peak in the third trimester, and a return to the standard volume after 6 weeks postpartum.^13^^,^^14^^,^^15^ Alterations in plasma metabolic profiles from pregnancy to the end of delivery in normal pregnancy are considered a physiological change.^16^ According to our results, tryptophan was significantly increased by 30% in postpartum plasma compared to the plasma during pregnancy (FDR q-value <0.001), which was consistent with a previous study.^26^ In contrast, pregnant women have lower fasting concentrations for most amino acids, and almost all amino acids in the amniotic fluid decline significantly with gestational age.^18^^,^^27^ A cohort study of pregnant and non-pregnant women showed strong positive associations of pregnancy with amino acids including alanine, phenylalanine, and histidine, and negative associations of pregnancy with glutamine, glycine, valine, and tyrosine.^28^ Our results showed that 18 amino acids (alanine, arginine, asparagine, cysteine, glutamic acid, glutamine, glycine, histidine, isoleucine, leucine, lysine, methionine, phenylalanine, proline, serine, tryptophan, tyrosine, and valine) significantly increased, but one, threonine, decreased after childbirth (Table S5 in the supplemental information). In the current study, the most significantly changed metabolic component, i.e., aminoacyl-tRNA synthetases (ARSs), was found in both the control and PPD groups after childbirth. In a diseased state, ARSs inhibit amino acid synthesis, in which protein accumulation is not translated to regulate the corresponding tRNA.^29^ However, pregnancy and childbirth are not states of ill health, indicating that common changes in ARSs and other enriched metabolic pathways, such as arginine biosynthesis and valine, leucine, and isoleucine biosynthesis, constitute a physiological response in both women with and without PPD. Overall, the effect of plasma recovery behavior in each multiparous woman may change the statistical significance of postpartum plasma research, explaining the varied results reported by previous metabolic studies on PPD.^6^^,^^7^^,^^8^^,^^9^^,^^10^^,^^11^^,^^12^ The current study suggests that research on PPD should consider the plasma recovery behavior of individuals.

Comparing the control and PPD groups, metabolic disturbances seemed to occur from mid-late pregnancy to 1 month postpartum resulted in the metabolic category, TCA cycle. The TCA cycle is the final common pathway for the oxidation of carbohydrates, proteins, and lipids, generating nearly two-third of total energy.^30^ Furthermore, based on the STITCH analysis, those metabolites interact with glucose, sucrose, oleic acid, and pyruvic acid, which are primary sources for the TCA cycle.^31^ A dysfunction of the TCA cycle suggests that metabolic disturbances in women with PPD might be linked to the underlying behavioral and cognitive development of the offspring,^4^^,^^32^ such as the development of autism spectrum disorder.^33^ However, the metabolic disturbances underlying molecular mechanisms are unclear and need to be further characterized.

We also identified metabolites that were significantly different between the plasma of pregnant and postpartum women by drawing direct comparisons between the plasma of pregnant and postpartum women without (EPDS ≤2, n = 222) and with PPD (EPDS ≥9, n = 209). Increased cysteine and decreased cytosine levels in the plasma of pregnant women with PPD showed significant changes as compared with the control (FDR q-value <0.05; Table S3 in the supplemental information). Cysteine is an essential proteinogenic amino acid that accounts for 2% of all structural proteins, such as cell membranes and myelin sheaths around neurons, structures that protect neurons from oxidative stress and harsh environmental conditions.^34^ Another significantly decreased metabolite during pregnancy, i.e., cytosine, is one of the four main nucleotide bases of DNA and RNA and regulates pyrimidine nucleotide biosynthesis. Pyrimidine metabolism has recently been implicated in antidepressant treatment response^35^ and is disrupted in female college students with depression.^36^ Microarray analysis has revealed that pyrimidine metabolism plays a crucial role in the pathophysiology of major depression.^37^ Machine learning also revealed cytosine as a strong biomarker for PPD prediction during pregnancy, a partially key molecule for the development of PPD. In addition, two postpartum metabolites, phenylacetic acid (PAA) and monostearin, were decreased in the PPD group compared with the control group. PAA is a major deaminated metabolite of phenylethylamine in mammals and is associated with depression. For instance, decreased PAA was confirmed in the cerebrospinal fluid, urine, and plasma of patients with depressive illness.^38^^,^^39^^,^^40^ Another significantly decreased postpartum metabolite, monostearin, which is commonly used as a food additive, has been reported to be changed in previous metabolic studies but without information on its biological function, which requires further research.

Through machine learning we identified erythrulose as a risk factor for PPD, which showed inverse alterations in women with PPD and significantly increased in pregnancy but decreased postpartum. Erythrulose is a ketotetrose, belongs to the ketose family, and is involved in “Tetrose metabolism.”^41^^,^^42^ Tetrose is considered an enzyme inhibitor in the glycolysis pathway,^43^ inhibiting glucose breakdown and producing energy in the form of ATP for the TCA cycle. Release of ATP is impaired in patients with major depression^44^ and murine models of depression,^45^ which can most likely be because of the association of erythrulose with oxidative phosphorylation or due to ATP production defects in women with PPD.

In conclusions, the current study determined physiological changes in plasma metabolome in normal pregnancy from mid-late gestation to 1 month postpartum. Our findings have important implications for metabolism studies in both pregnant and postpartum plasma, which observed significantly different metabolic changes during pregnancy and postpartum. Based on our findings, we posited methods, provided a PPD-related metabolic pathway from pregnancy, and proposed predictable biomarkers for early prediction of PPD warrant additional investigations.

### Limitations of the study

The strengths of the present study include the large, paired plasma samples (431 women) during mid-late gestation and at 1 month postpartum. Our results produced more accurate metabolic profiles compared to that reported in previous OPLS-DA and PCA research. Furthermore, as an exploratory research, our analyses in the control group, which showed EPDS scores of <3 enabled us to assess robust metabolic changes that underlie PPD development. The comparison of the control group with EPDS scores <3 and women with PPD could lead to ungeneralizable results because of the expected extreme differences in clinical profiles between these groups. Future studies in this field should choose a control group with a more similar clinical profile to women with PPD to address this. Our data were obtained from a single region Japanese cohort. The results do not validate the varying demographic features with an independent cohort in different regions (countries) and, thus, cannot be generalized. Furthermore, machine learning across independent cohorts generally improves the prediction capabilities for disease, as overfitting can occur when training a model based on a single cohort. The information on maternal pregnancy complications was not collected in the current study that should be conducted in future studies. Several important clinical information in the current study should be carefully discussed in the future. Above have implications for the interpretation of findings and the generalizability of the results.

## STAR★Methods

### Key resources table

**Table:** 

REAGENT or RESOURCE	SOURCE	IDENTIFIER
**Chemicals, peptides, and recombinant proteins**		

N-Methyl-N-(trimethylsilyl)trifluoroacetamide	Sigma-Aldrich	Cat# 69479-25ML
Human 2-isopropylmalic acid-3TMS	Sigma-Aldrich	Cat# 333115-100MG

**Deposited data**		

iMorp: Metabolome 2022	jMorp	https://jmorp.megabank.tohoku.ac.jp/

**Software and algorithms**		

SPSS version 22.0	IBM	RRID:SCR_002865
SIMCA ver. version 17.0.1	Sartorius	RRID:SCR_014688
JMP Pro 15.0.0	SAS Institute	RRID:SCR_014242

**Other**		

Gas Chromatography Mass Spectrometer-TQ8040	Shimadzu	GCMS-TQ8040
Autosampler system	Shimadzu	AOC6000

### Resource availability

#### Lead contact

Further information and requests for resources and reagents should be directed to and will be provided by the lead contact, Zhiqian Yu (yu_zhiqian@med.tohoku.ac.jp).

#### Materials availability

This study did not generate new unique reagents.

### Experimental model and subject details

#### Ethical approval

The study was approved by the Ethical Research Committee at Tohoku University Graduate School of Medicine (2013-1-103-1, 2021-1-266). We obtained written informed consent from all participants who agreed to participate in the TMM BirThree Cohort Study. This report was conducted according to the STROBE guidelines for reporting observational studies in epidemiology.^46^

#### Participants and study settings

This analysis targeted plasma metabolic profiles in mid-late gestation and 1-month postpartum in 431 women selected using the Japanese version of the Edinburgh Postnatal Depression Scale (EPDS) from the Tohoku Medical Megabank Project Birth and Three-Generation cohort study. Women with PPD (EPDS ≧ 9; n = 209; age: 30.89 ± 4.95) and without PPD (controls, EPDS ≦ 2; n = 222; age: 31.46 ± 3.73) were selected and matched for age and gestational weeks (24th–27th weeks). This observational case-control study population is based on the Tohoku Medical Megabank Project (ToMMo) Birth and Three-Generation Cohort Study (TMM BirThree Cohort Study), a general population-based prospective cohort study that began in 2011.^47^^,^^48^ The cohort studies of ToMMo were objective with regard to monitoring individual health status and the implementation of suitably timed interventions after the Great East Japan Earthquake and subsequent tsunami by Tohoku University ToMMo and Iwate Medical University Iwate Tohoku Medical Megabank Organization (IMM). The information for mothers and newborns used in this study was collected and cleaned until July 2021.

#### Protocol for assessing postpartum depressive symptoms and plasma sample selection

The Japanese version of the Kessler Psychological Distress Scale (K6) and the EPDS score were collected at two time points: at 24^th^-27^th^ weeks of gestation and fourth-fifth weeks postpartum. The K6 and EPDS were employed to evaluate mental status during pregnancy and postpartum, respectively, and blood samples were collected.

The K6 is a short screening instrument for mental illness in the general population. K6 scores of equal to and lower than 4 points are classified as non-psychological distress pregnancy; in the current study, a K6 score of 13 points or higher was considered to indicate severe psychological distress.^20^^,^^49^ Furthermore, the Japanese version of the K6 consists of six questions that have been validated previously, and scores were divided into four groups (normal: 0–4, low: 5–9, moderate: 10–12, high: 13–24).^50^^,^^51^

Western studies use EPDS scores of 13 or higher to distinguish women with PPD.^52^ In 1996, Okano et al. introduced EPDS scoring to Japan and set the cutoff score to 8/9 for screening PPD.^53^ This cutoff difference is to account for the documented tendency for Japanese women to be less expressive of their feelings than Western women^54^ and has been validated and subsequently used in Japan.^55^^,^^56^^,^^57^ Herein, A Japanese version of the EPDS was used in the present study, and women with an EPDS score equal to or greater than 9 is considered PPD. To obtain the most distinguishable metabolic profiling for women with PPD, in this study, women without PPD (control) was indicated by an EPDS score equal to or lower than 2.

In total, out of the 8,714 mothers in the TMM BirThree Cohort Study, 14.25% of mothers had EPDS scores equal to or greater than 9, a result similar to those of a previous meta-analysis on the prevalence of PPD in Japan that reported 14.3% of mothers with EPDS scores ≥9.^58^ The number of individuals in each EPDS score category is summarized in Figure S1A. Among the cohort datasets, whereas 6,206 were confirmed to have both K6 (24^th^–27^th^ weeks pregnancy) and EPDS scores. The required sample size was calculated using G∗Power 3.1.9.7.^59^ Assuming an alpha level of 0.05%, 95% power, a medium effect size (Cohen’s *f* = 0.25), 2 groups (EPDS ≤2 and EPDS ≥9), and 3 covariates (BMI, smoking and alcohol drinking), the desired sample size was 210. Therefore, this study was able to meet the sample size needed to test this hypothesis with 500 participants.

Based on the above required sample size and EPDS cutoff, 250 women with an EPDS score less than or equal to 2 (EPDS ≤2) and an EPDS score greater than or equal to 9 (EPDS ≥9) were randomly selected and age matched; plasma samples from these women in pregnancy and postpartum as control and PPD groups, respectively, were used for GC-MS analyses. Furthermore, as exploratory research, our analyses in healthy women (control group) with EPDS scores less than three intended to determine more robust metabolic changes at the systemic level that underlie PPD development. The similarity of the mean age at delivery of the PPD and control groups reflected the initial age matching (p = 0.139). Details regarding mental status in mid-late pregnancy and postpartum, body mass index (BMI) in early gestation (5^th^–13^th^ weeks) and at 1 month postpartum, previous births, body weight and height of newborns, smoking and drinking during pregnancy, blood chemical examination, urine test, are recorded. Preterm births (less than 37 weeks' gestation) were excluded in the current data.

### Method details

#### Plasma sample collection and preparation

Plasma samples were collected during pregnancy and postpartum after prenatal K6 or postpartum EPDS evaluation. The plasma was prepared as previously described.^60^ In brief, blood was collected using Venoject II tubes containing EDTA-2Na (Terumo Corporation) and centrifuged at 2,330 × g for 10minat 4°C. The plasma was transferred to a liquid handling machine (Freedom EVO, Tecan) and dispensed into MATRIX 1.0-mL 2D barcoded screw-cap tubes (Thermo Scientific). Approximately 700 μL plasma in each tube aliquoted by TMM Biobank ID was stored at −80°C. For GC-MS analysis, 50 μL plasma was automatically dispensed by the VERSETTE system (Thermo Fisher, USA). A GCMS-TQ8040 (Shimadzu, Kyoto, Japan) was utilized for GC-MS.

#### GC-MS performance and metabolite analysis

In the current study, GC-MS was used in the TMM BirThree cohort. The procedure for plasma sample preparation using an automatic pretreatment system has been previously described.^61^^,^^62^^,^^63^ Plasma metabolites were extracted by a robotic system (Microlab STARlet robot system, Hamilton, Reno, NV) using as the extraction solvent chloroform (KANTO CHEMICAL, Tokyo)/methanol (KANTO CHEMICAL, Tokyo)/water (Milli-Q, Millipore, Burlington)/internal standard (IS) solution (0.5 mg/mL containing 2-isopropylmalic acid; Sigma, St. Louis, MO) at ½.5/1/0.18 (v/v/v/v) (260 μL). The extracted solution was lyophilized with a freeze-drying system (FDS-1000, EYELA, Tokyo), and methoxyamine hydrochloride (Sigma) in pyridine (Tokyo Chemical Industry Co. Ltd., Tokyo) (20 mg/mL, 80 μL) was added. The sample was homogenized in an ultrasonic bath (UT-10, Sharp, Osaka) and agitated at 1200 rpm for 90minat 30°C (Eppendorf, Hamburg, Germany). After centrifugation at 16,000 × g for 3minat 4°C, the supernatant (40 μL) was transferred to a vial (2.0 mL) (Shimadzu GLC, Tokyo) and placed in an autosampler system (AOC6000, Shimadzu, Kyoto). N-Methyl-N-(trimethylsilyl)trifluoroacetamide (MSTFA, GL Science, Saitama) (20 μL) was added, and the sample was shaken at 37°C for 30 min, followed by GC-MS/MS (1 μL) using previously described analytical conditions.^63^

The internal standard (IS) human 2-isopropylmalic acid was purchased from Sigma-Aldrich (St. Louis, MO, USA), and 5 μL of 1.0 mg/mL IS was added to each plasma sample, which was subjected to GC-MS to correct for intrabatch and interbatch differences.^63^^,^^64^ The peak height of each quantified ion was identified with Smart Metabolites Database (Shimadzu), and the signal (area ratio) of each metabolite (170 metabolites/sample) was normalized and calculated as analyte area/IS area (in each sample) using TraversMS software (Reifycs, Tokyo, Japan) (Figure S1A in supplemental information).

#### In silico analysis of plasma metabolic networks

MetaboAnalyst’s biomarker analysis module was used to identify the potential metabolites for PPD prediction (https://github.com/xia-lab/MetaboAnalystR).^25^ In detail, the multivariate ROC curve exploratory analysis in MetaboAnalyst aims to evaluate the performance of biomarker models created through automated important feature identification. Using balanced sub-sampling, ROC curves are generated by Monte Carlo cross-validation (MCCV). In each MCCV, ⅔ of the samples are used to evaluate feature importance, and the remaining ⅓ validate the models created with the first step. We used 10-fold CV to justify the performance of classifiers by using random forest. The top-ranking features (max top 100) in importance are used to build the biomarker classification models after repeated calculation of the performance and confidence intervals of the model. The plot description by selected frequency (%) will indicate the most important features of a selected model ranked from most to least important.

A bioinformatics tool was employed to visualize the metabolic networks and pathways. Metabolite set enrichment analysis (MSEA)^65^ and Small Molecule Pathway Database (SMPDB; http://smpdb.ca)^66^ were used to identify and interpret patterns of metabolic changes and pathways in a biologically meaningful context. In addition, metabolic networks related to the most abundant metabolites were analyzed using Metscape version 3.1.3, which is a plug-in for Cytoscape version 3.9.1 (http://www.cytoscape.org/),^67^ to examine relationships among metabolites (and with other omics, enzymes and genes) and biological functions. Metabolite-protein interactions network was generated using the Search Tool for Interactions of Chemicals (STITCH; database, version 5.0 (http://stitch.embl.de/).^23^ Metabolic pathway identification was performed using Kyoto Encyclopedia of Genes and Genomes.^68^

### Quantification and statistical analysis

Among 500 women, we excluded those with multiple births (control group, n = 0; PPD group, n = 0), those who withdrew consent (control group, n = 0; PPD group, n = 5), those with hypertensive pregnancy disorders who were diagnosed later (control group, n = 18; PPD group, n = 32), and those with missing GC-MS data of over 50% for either the pregnancy or postpartum plasma sample (control group, n = 10; PPD group, n = 4). Paired GC-MS data for pregnancy and postpartum samples from women with PPD (EPDS ≥9, n = 209) or without PPD (EPDS ≤2, n = 222) were analyzed.

The normalized signal of the automatically identified plasma metabolites was imported into SIMCA version 17.0.1 (Umetrics, Umeå, Sweden) and adjusted by unit variance (UV) scaling. Normalized data principal component analysis (PCA) was first performed as unsupervised clustering to identify similarity or differences between sample profiles. After the grouping, trends and outliers were revealed from a scatterplot; orthogonal partial least squares discriminant analysis (OPLS-DA) was also used for discrimination. To assess whether plasma metabolites can distinguish among metabolites, discriminating metabolites were suggested by OPLS-DA from one predictive and two or more orthogonal components. The quality of the OPLS-DA models was described by the cumulative modeled variation in the X matrix R^2^X(cum), the cumulative modeled variation in the Y matrix R^2^Y(cum), where R^2^Y(cum) is defined as the proportion of variance in the data explained by the models and indicates goodness of fit, and cross-validated predictive ability *Q*^2^(cum) values. OPLS-DA models were rejected if they presented complete overlap of *Q*^2^ distributions [*Q*^2^(cum) < 0.5] or low classification rates [R^2^(cum) < 0.65].^69^

Comparison of two groups was performed using ANCOVA with covariates including age, BMI, smoking, and K6 scores. Student’s paired or unpaired t-test was applied to determine the significantly changed metabolites in GC-MS data following the failure detection rate (FDR), up to 95% by SPSS Statistics R plug-in (version 22.0. IBM Japan, Tokyo, Japan). Differences among predictive metabolites were deemed statistically significant using one-way ANOVA for multiple comparisons and Tukey’s correction in SPSS. To determine diagnostic effectiveness, significantly different candidate metabolites were analyzed with nonparametric receiver operating characteristic (ROC) curves in JMP Pro 15.0.0 (SAS Institute, Cary, North Carolina, USA).

## Data Availability

• The samples and metabolomics data supporting the current study are available from Tohoku University Tohoku Medical Megabank Organization (ToMMo; https://www.megabank.tohoku.ac.jp/english/sample/) and the Japanese MultiOmics Reference Panel (Jmorp; https://jmorp.megabank.tohoku.ac.jp/). • This paper does not report original code. • Any additional information required to reanalyze the data reported in this paper is available from the lead contact upon request.

## Supplemental information

Document S1. Figures S1–S5 and Tables S4–S9

Table S1. The clustering of plasma metabolic profiles, related to Figure 2B Plasma metabolic profiles during mid-late gestation and at one month postpartum between women without postpartum depression (PPD) (EPDS ≤ 2; n = 222) and women with PPD (EPDS ≥ 9; n = 209).

Table S2. Metabolic profiles after 1 month delivery, related to Figure 3A Maternal plasma metabolic changes at 1 month postpartum between women without (EPDS ≤ 2; n = 222) and with postpartum depression (EPDS ≥ 9; n = 209).

Table S3. Metabolic profiles during pregnancy, related to Figure 3B Maternal plasma metabolic changes at mid-late gestation between women without postpartum depression (PPD) (EPDS ≤ 2; n = 222) and women with PPD (EPDS ≥ 9; n = 209).

Table S10. Potential biomarkers of the training dataset, related to Figure 5C Rank on the percentage of the frequency selected in the multivariate ROC curve-based model using Monte-Carlo cross-validation based on the random forest algorithm.

Table S11. Potential biomarkers of the test dataset, related to Figure 5C Rank on the percentage of the frequency selected in the multivariate ROC curve-based model using Monte-Carlo cross-validation based on the random forest algorithm.
