# Supplementary material for: Plasma metabolic disturbances during pregnancy and postpartum in women with depression
Source: iScience. 2022 Nov 24;25(12):105666. doi: 10.1016/j.isci.2022.105666 (PMC9732390; doi:10.1016/j.isci.2022.105666)
Supplement: Document S1. Figures S1–S5 and Tables S4–S9 [file mmc1.pdf]

iScience, Volume *25*

Supplemental information

Plasma metabolic disturbances during pregnancy and postpartum in women with depression

Zhiqian Yu, Naomi Matsukawa, Daisuke Saigusa, Ikuko N. Motoike, Chiaki Ono, Yasunobu Okamura, Tomomi Onuma, Yuta Takahashi, Mai Sakai, Hisaaki Kudo, Taku Obara, Keiko Murakami, Matusyuki Shirota, Saya Kikuchi, Natsuko Kobayashi, Yoshie Kikuchi, Junichi Sugawara, Naoko Minegishi, Soichi Ogishima, Kengo Kinoshita, Masayuki Yamamoto, Nobuo Yaegashi, Shinichi Kuriyama, Seizo Koshiba, and Hiroaki Tomita

Supplemental Information

Supplementary Figures

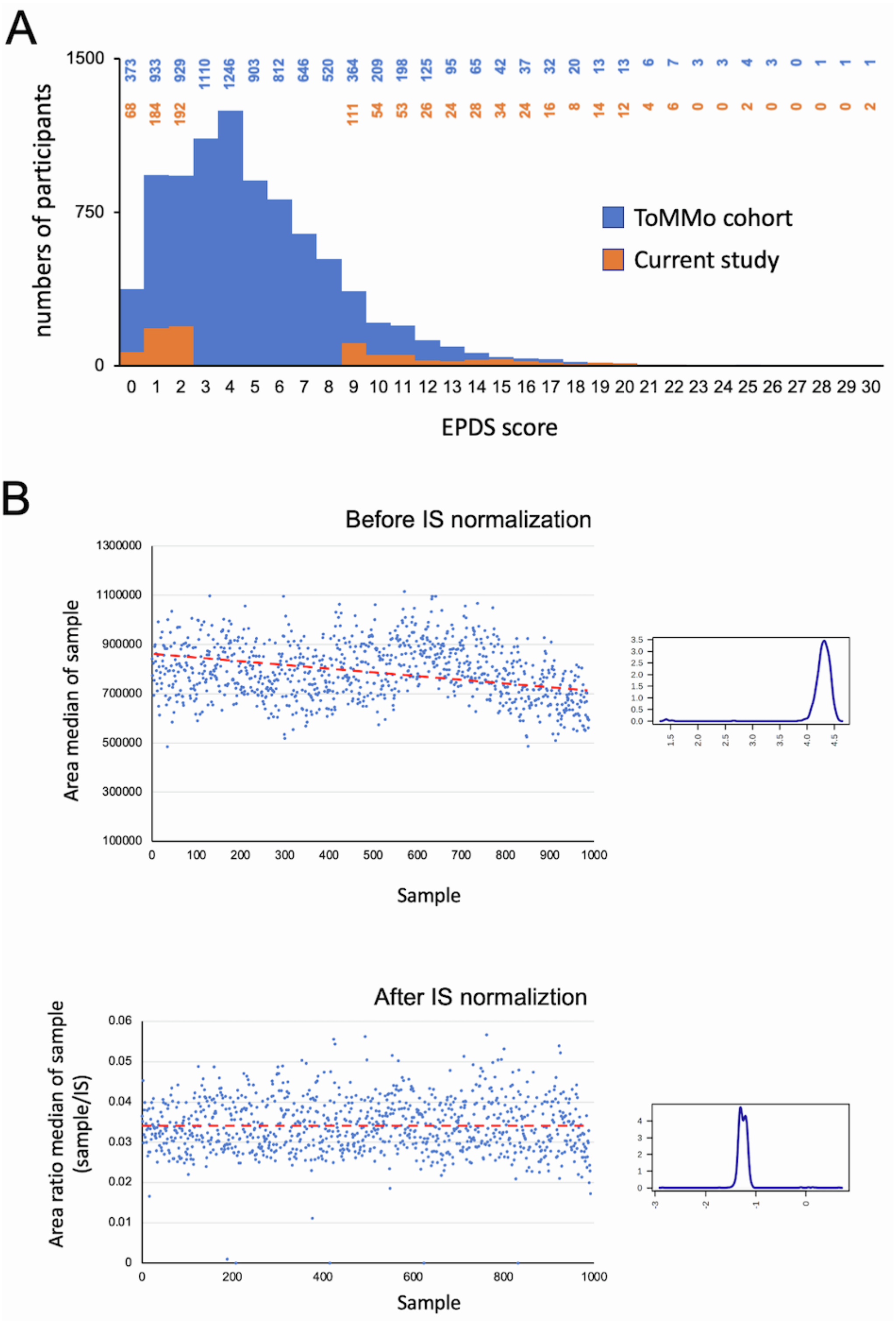

Figure S1. The distribution of EPDS scores and normalization of gas chromatography-mass spectrometry data, Related to Figure2A.

(A) The distribution of individuals in each EPDS score category from 0–30 was summarized as histograms for the ToMMo cohort (n = 8,714) and the current study in paired subjects of metabolic research (control: n = 222; PPD: n = 209). Blue columns represent the ToMMo cohort. Orange columns represent the current study. Numbers indicate the individuals in each EPDS score category. EPDS, the Japanese version of the Edinburgh Postnatal Depression Scale. (B) Unnormalized (upper) and internal standard normalized (lower) data of the median metabolite signal intensities from the participants. IS, internal standard.

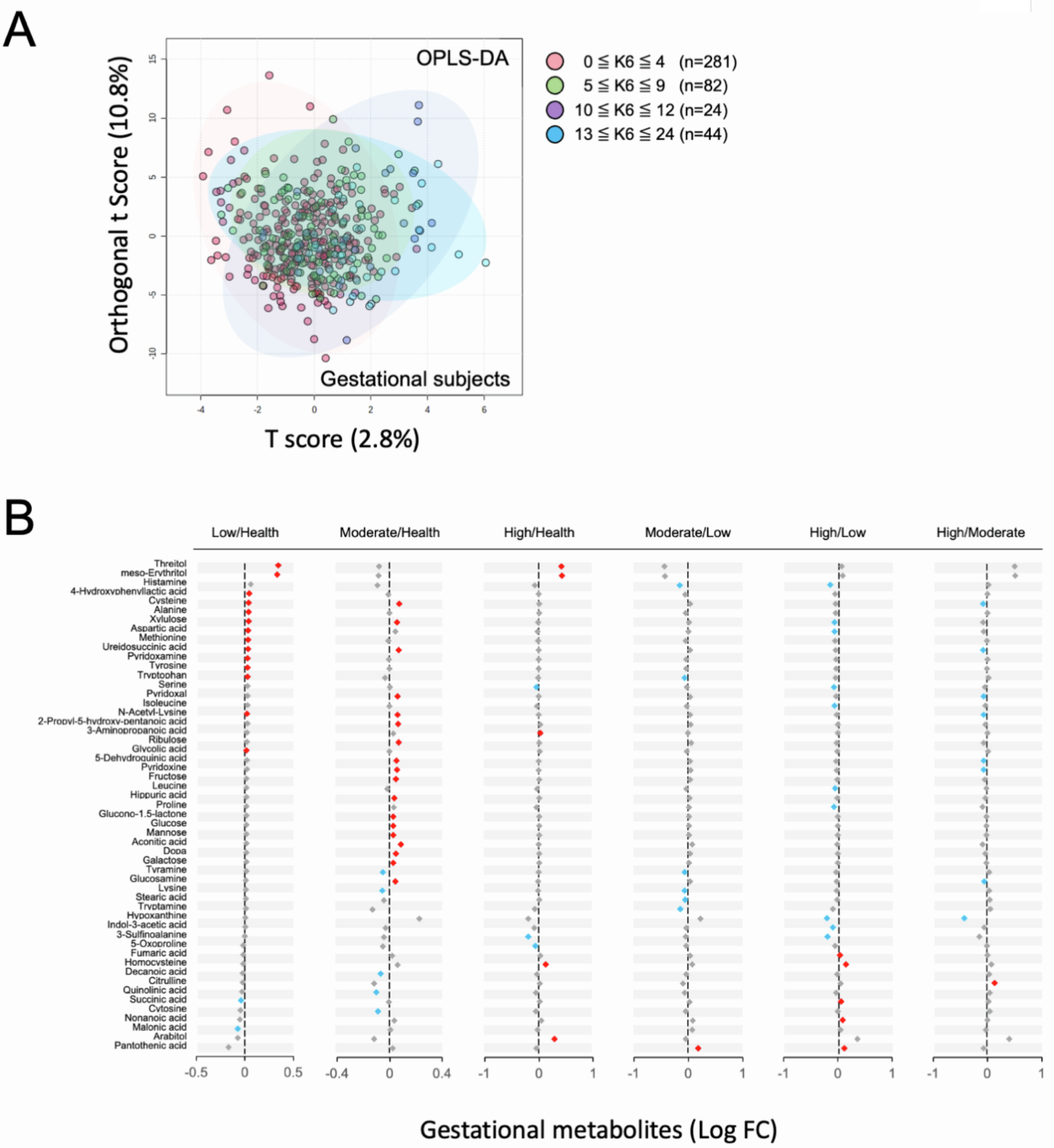

Figure S2. Effects of psychological distress on gestational metabolites during pregnancy, Related to Figure 2B.

(A) Orthogonal Projections to Latent Structures Discriminant Analysis (OPLS-DA) generating discrimination of gestational metabolic groups by K6 (the Japanese version of the Kessler Psychological Distress Scale) scores. (B) The forest plot shows the average log fold change (FC) of metabolites that changed in each K6 group comparison: Health, 0 ≤ K6 ≤ 4 (n = 281); Low, 5 ≤ K6 ≤ 9 (n = 82); moderate, 10 ≤ K6 ≤ 12 (n = 24); and High, 13 ≤ K6 ≤ 24 (n = 44). Red circles indicate log FC > 0 and *P* <0.05 (Student's t-test), blue circles indicate log FC < 0 and *P*

<0.05 (Student's t-test), and the gray color indicates P > 0.05 (Student's t-test). There is no significantly different metabolite after FDR correction (q value > 0.05).

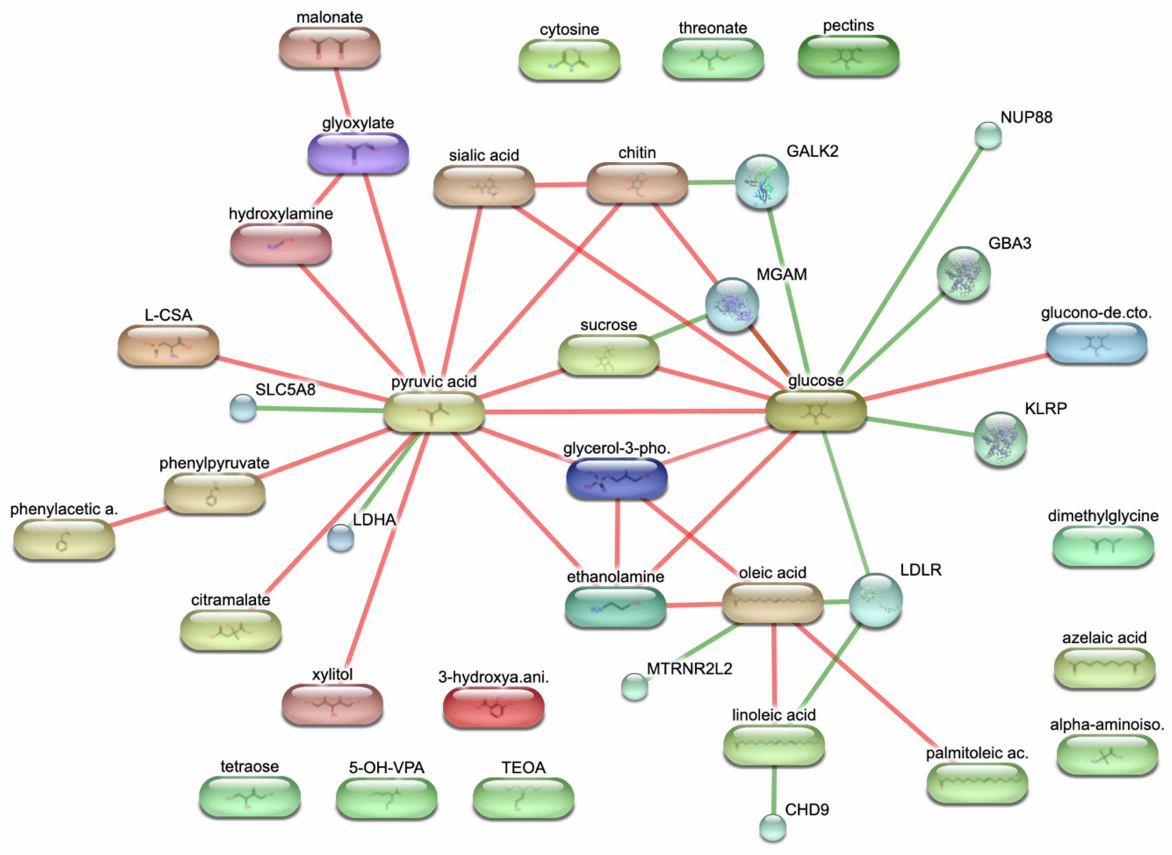

Figure S3. Metabolomic network potentially involved in the development of postpartum depression from pregnancy, Related to Figure 4C.

The network of 33 of 37 nonoverlapped metabolites (four metabolites, 2-Aminopimelic acid, margaric acid, monostearin, and N-butyrylglycine were not recognized by KEGG) using the STITCH Network nodes are colored if they are directly linked to the input with a higher iteration/depth. Lines between nodes (edges) indicate predicted functional links, where thicker lines represent stronger associations; Protein-protein interactions are shown in grey, chemical-protein interactions in green, and interactions between chemicals in red.

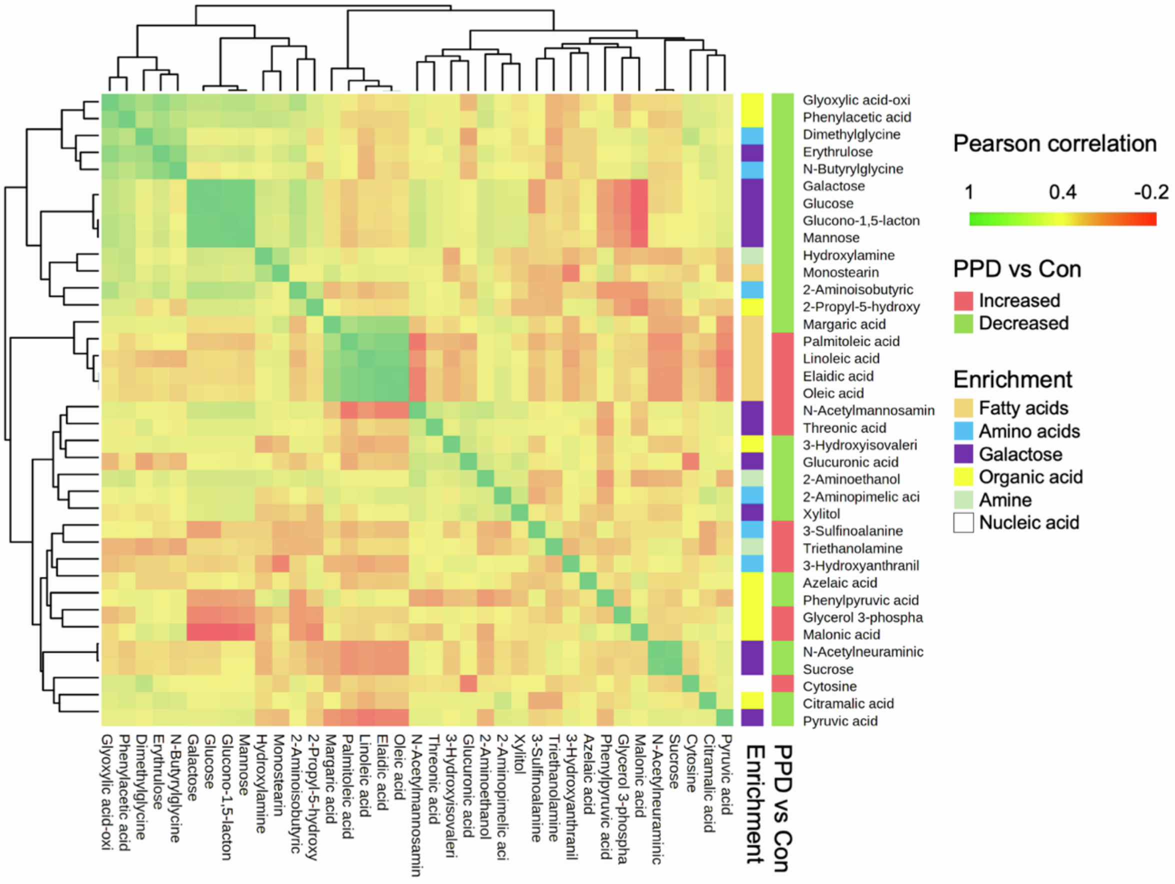

Figure S4. Different metabolic changes between women with and without postpartum depression, Related to Figure 4C.

Correlation matrix colored by the Pearson correlation coefficient of 37 metabolic different recovery rates (RR) from pregnancy to postpartum in women with and without postpartum depression (PPD). Con, women without PPD. PPD vs Con, RR of PPD/RR of Con.

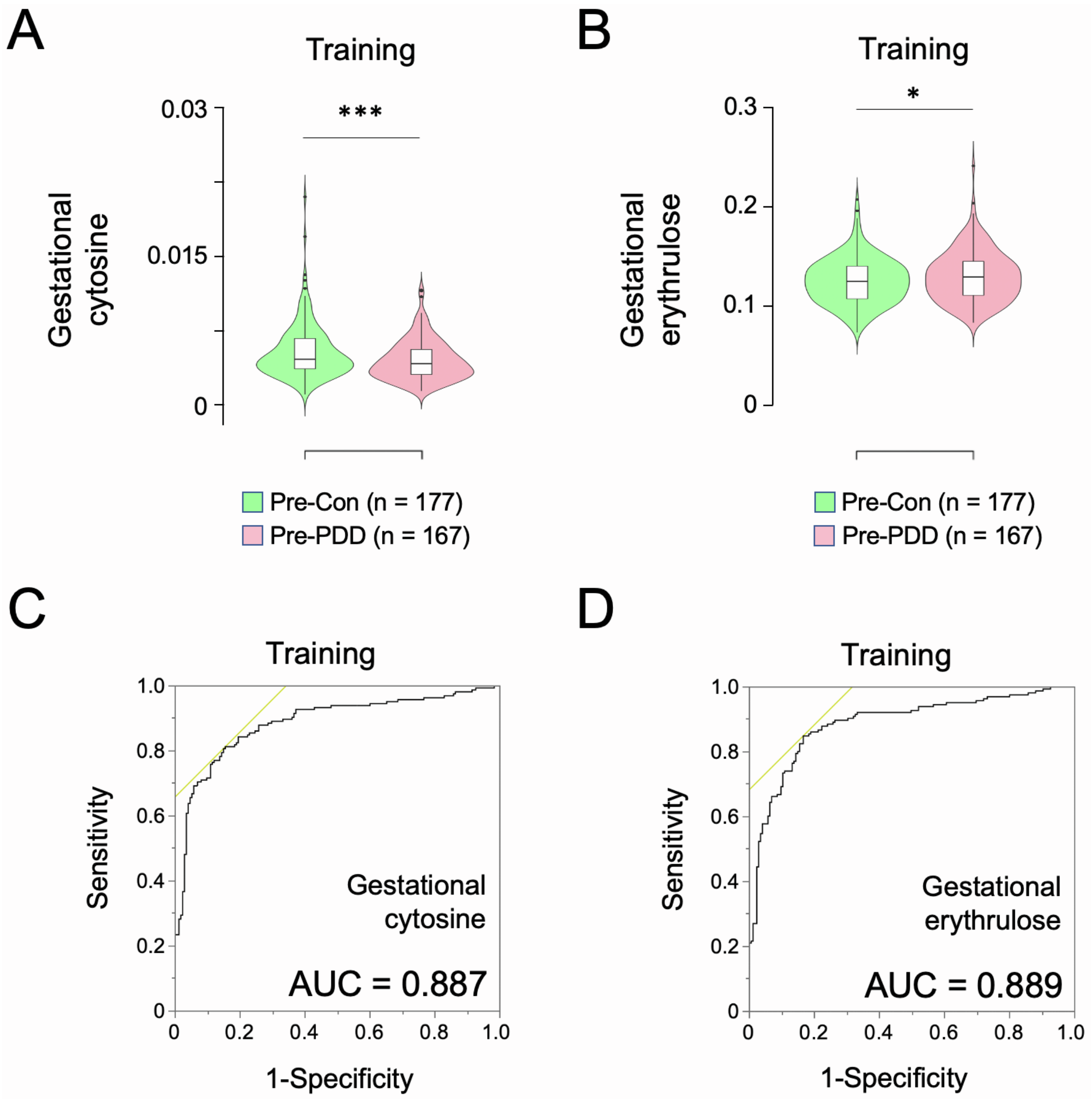

Figure S5. Gestational metabolic changes and ROC analysis in the training dataset, Related to Figure 5B.

(A) Violin plots of plasma cytosine levels at pregnancy period were significantly decreased in women with PPD (n = 167) compared with control (n = 177). ($F_{1, 342}$ = 11.212, *P* = 0.0009). Violin plots show the frequency (density plot width) of each group with 95% confidence intervals, interquartile ranges, and median values. Pre-PPD vs. Pre-Con *P* <0.001. Statistical significance measured by ANCOVA with covariates including age, BMI, smoking, and K6 scores. (B) Violin plots of plasma erythrulose levels at pregnancy period were significantly increased in women with PPD (n = 167) compared with control (n = 177). Pre-PPD vs. Pre-Con, *P* <0.05. Statistical significance measured by ANCOVA with covariates including age, BMI, smoking, and K6 scores.

(C) ROC analysis of cytosine levels in the training dataset at pregnancy period based on the EPDS cutoff score of 2/9 (AUC = 0.887, OR = 0.085, 95 % CI 0.007 - 0.810). (D) ROC analysis of erythrulose levels in the training dataset at pregnancy period based on the EPDS cutoff score of 2/9 (AUC = 0.889, OR = 6.518, 95 % CI 1.153 - 47.327). When the K6 score, which was significantly higher in the PPD than in the control group was excluded from the covariates, the discriminative values were higher than 0.6 for cytosine (AUC = 0.622, OR = 0.04, 95% CI 0.005 – 0.274) and for erythrulose (AUC = 0.618, OR = 3.851, 95% CI 1.087 – 17.533). EPDS, the score of the Japanese version of the Edinburgh Postnatal Depression Scale. PPD, postpartum depression; Pre-Con, plasma metabolites in pregnancy in controls; Pre-PPD, plasma metabolites in pregnancy in women without PPD. * P <0.05, *** P <0.001. K6: The Japanese version of the Kessler Psychological Distress Scale. EPDS: The Japanese version of the Edinburgh Postnatal Depression Scale.

Supplementary Tables

Table S4. Maternal plasma metabolic changes at mid-late gestation among the healthy women and women with low, moderate, and high levels of psychological distress (P <0.05), Related to Figure 2B.

Metabolite	Low/Health (FC)	Moderate/Health (FC)	High/Health (FC)	Moderate/low (FC)	High/Low (FC)	High/Moderate (FC)
Threitol	0.350	-0.081	0.424	-0.431	0.074	0.505
meso-Erythritol	0.339	-0.086	0.429	-0.425	0.090	0.515
Histamine	0.063	-0.096	-0.072	-0.159	-0.135	0.024
4-Hydroxyphenyllactic acid	0.047	-0.009	0.001	-0.056	-0.046	0.010
Cysteine	0.042	0.075	0.005	0.032	-0.037	-0.069
Alanine	0.041	-0.003	0.008	-0.045	-0.033	0.011
Xylulose	0.041	0.058	-0.014	0.017	-0.055	-0.072
Aspartic acid	0.038	0.042	-0.018	0.005	-0.056	-0.061
Methionine	0.037	-0.011	-0.012	-0.047	-0.049	-0.001
Ureidosuccinic acid	0.033	0.068	0.000	0.035	-0.033	-0.068
Pyridoxamine	0.031	-0.004	0.003	-0.035	-0.028	0.007
Tyrosine	0.030	-0.001	0.000	-0.031	-0.030	0.001
Tryptophan	0.029	-0.035	-0.006	-0.064	-0.035	0.029
Serine	0.026	0.001	-0.042	-0.025	-0.068	-0.043
Pyridoxal	0.025	0.060	-0.006	0.035	-0.031	-0.066
Isoleucine	0.025	-0.004	-0.031	-0.029	-0.055	-0.026
N-Acetyl-Lysine	0.025	0.062	0.004	0.037	-0.020	-0.057
2-Propyl-5-hydroxy-pentanoic acid	0.024	0.063	0.023	0.039	-0.001	-0.041

3-Aminopropanoic acid	0.023	0.024	0.029	0.001	0.006	0.004
Ribulose	0.023	0.069	0.002	0.047	-0.021	-0.068
Glycolic acid	0.020	0.000	0.017	-0.020	-0.003	0.017
5-Dehydroquinic acid	0.019	0.054	-0.009	0.034	-0.028	-0.063
Pyridoxine	0.019	0.056	-0.004	0.037	-0.023	-0.060
Fructose	0.018	0.046	0.002	0.028	-0.016	-0.045
Leucine	0.017	-0.020	-0.034	-0.037	-0.051	-0.014
Hippuric acid	0.017	0.036	0.001	0.019	-0.016	-0.035
Proline	0.017	0.031	-0.047	0.014	-0.064	-0.078
Glucono-1,5-lactone	0.015	0.030	0.008	0.014	-0.007	-0.021
Glucose	0.015	0.029	0.008	0.014	-0.006	-0.020
Mannose	0.014	0.027	0.008	0.012	-0.006	-0.018
Aconitic acid	0.014	0.086	-0.003	0.072	-0.017	-0.089
Dopa	0.014	0.048	0.007	0.034	-0.007	-0.041
Galactose	0.014	0.027	0.009	0.014	-0.005	-0.019
Tyramine	0.014	-0.052	-0.010	-0.065	-0.023	0.042
Glucosamine	0.013	0.042	-0.013	0.030	-0.026	-0.055
Lysine	0.010	-0.054	-0.013	-0.063	-0.022	0.041
Stearic acid	0.009	-0.046	0.001	-0.055	-0.008	0.047
Tryptamine	0.008	-0.132	-0.075	-0.141	-0.084	0.057
Hypoxanthine	0.006	0.224	-0.195	0.218	-0.201	-0.419
Indol-3-acetic acid	0.004	-0.033	-0.087	-0.037	-0.091	-0.054
3-Sulfinoalanine	0.000	-0.044	-0.187	-0.043	-0.187	-0.143
5-Oxoproline	-0.017	-0.055	-0.061	-0.038	-0.044	-0.006

Fumaric acid	-0.017	0.017	0.023	0.035	0.041	0.006
Homocysteine	-0.018	0.058	0.134	0.076	0.152	0.076
Decanoic acid	-0.023	-0.066	-0.031	-0.043	-0.009	0.034
Citrulline	-0.024	-0.118	0.019	-0.094	0.043	0.137
Quinolinic acid	-0.029	-0.101	-0.057	-0.072	-0.028	0.043
Succinic acid	-0.037	-0.006	0.023	0.031	0.060	0.029
Cytosine	-0.046	-0.090	-0.050	-0.044	-0.004	0.040
Nonanoic acid	-0.053	0.032	0.042	0.085	0.095	0.010
Malonic acid	-0.068	0.006	-0.018	0.075	0.051	-0.024
Arabitol	-0.071	-0.120	0.289	-0.049	0.360	0.409
Pantothenic acid	-0.168	0.020	-0.043	0.188	0.125	-0.063

Table S5. Significantly changed metabolites from mid-late gestation to 1 month postpartum in the control group (FDR q-value < 0.05) (EPDS ≤ 2; n = 222), Related to Figure 4A. Fold change, postpartum/pregnancy.

Metabolite	Fold change	t.stat	p.value	FDR
Ornithine	3.15	39.58	2.69E-102	4.58E-100
Glycine	2.27	29.92	1.16E-79	9.83E-78
Tyrosine	1.85	27.43	4.36E-73	2.21E-71
Pyridoxamine	1.91	27.40	5.20E-73	2.21E-71
Uric acid	1.60	26.79	2.38E-71	8.08E-70
Glycyl-Glycine	3.24	25.04	1.80E-66	5.09E-65
Dopa	1.59	24.82	7.64E-66	1.86E-64
Serine	1.74	23.74	1.01E-62	2.14E-61
Proline	2.08	21.58	2.54E-56	4.79E-55
Dihydroxyacetone	1.60	20.87	3.61E-54	6.14E-53
Phenylalanine	1.48	20.57	3.10E-53	4.80E-52
Methionine sulfone	3.12	20.34	1.55E-52	2.20E-51
Valine	1.48	19.95	2.45E-51	3.21E-50
Maleic acid	2.43	19.44	9.99E-50	1.14E-48
Glyceraldehyde	1.49	19.44	1.00E-49	1.14E-48
4-Hydroxyphenyllactic acid	1.92	19.12	9.42E-49	1.00E-47
Methylmalonic acid	1.55	18.95	3.40E-48	3.40E-47
Acetylglycine	2.23	18.85	6.67E-48	6.30E-47
Arginine	1.98	17.89	7.42E-45	6.64E-44
2-Hydroxyisobutyric acid	1.43	16.62	8.76E-41	7.45E-40
Urea	1.31	15.96	1.15E-38	9.33E-38
Isoleucine	1.52	15.46	4.94E-37	3.82E-36
4-Hydroxyproline	1.78	15.42	6.43E-37	4.75E-36
Indol-3-acetic acid	2.31	15.20	3.44E-36	2.44E-35
Glutamic acid	1.55	14.87	3.94E-35	2.68E-34
Leucine	1.46	14.84	5.09E-35	3.33E-34
Citrulline	2.18	14.58	3.58E-34	2.26E-33
Alanine	1.38	14.38	1.60E-33	9.72E-33
Creatinine	1.84	14.22	4.96E-33	2.91E-32
Tryptamine	2.24	14.01	2.54E-32	1.40E-31
1,5-Anhydro-glucitol	1.65	14.00	2.55E-32	1.40E-31
Kynurenine	1.64	13.87	7.13E-32	3.79E-31
Cysteine	1.57	13.74	1.89E-31	9.74E-31
Methionine	1.50	13.51	1.04E-30	5.22E-30
Lysine	1.36	13.45	1.53E-30	7.45E-30
Tyramine	1.35	13.21	9.69E-30	4.58E-29
Hypotaurine	1.86	13.17	1.27E-29	5.83E-29
Tryptophan	1.35	13.05	3.19E-29	1.43E-28
Isocitric acid	1.35	13.02	3.85E-29	1.68E-28
Arabinose	1.87	12.57	1.05E-27	4.48E-27
2-Ketoglutaric acid	1.36	12.55	1.26E-27	5.24E-27
Xylose	1.84	12.20	1.67E-26	6.78E-26
Cystine	1.55	12.15	2.29E-26	9.06E-26

Glycolic acid	1.19	11.83	2.52E-25	9.74E-25
Succinic acid	1.37	11.80	3.10E-25	1.17E-24
5-Oxoproline	1.41	11.62	1.17E-24	4.31E-24
Phosphoric acid	1.17	11.53	2.24E-24	8.10E-24
Lyxose	1.79	11.42	4.88E-24	1.73E-23
3-Aminopropanoic acid	1.40	11.21	2.17E-23	7.53E-23
N-Acetylglutamine	1.42	11.01	9.21E-23	3.13E-22
Ethylmalonic acid	1.35	10.96	1.30E-22	4.34E-22
2-Hydroxyglutaric acid	1.19	10.74	6.17E-22	2.02E-21
Glutaric acid	1.85	10.61	1.58E-21	5.07E-21
2-Aminoadipic acid	1.52	10.38	8.34E-21	2.63E-20
3-Methyl-2-oxovaleric acid	1.21	10.08	6.71E-20	2.07E-19
2-Ketoisocaproic acid	1.23	9.42	6.43E-18	1.95E-17
Ribonic acid	1.32	8.61	1.37E-15	4.08E-15
Malic acid	1.38	8.24	1.49E-14	4.37E-14
Dihydroorotic acid	1.48	8.14	2.88E-14	8.29E-14
Homocysteine	1.74	7.94	1.02E-13	2.88E-13
Glutamine	1.40	7.91	1.21E-13	3.39E-13
2-Deoxy-glucose	1.42	7.79	2.60E-13	7.14E-13
2-Hydroxyisovaleric acid	1.17	7.66	5.81E-13	1.57E-12
Threonine	0.84	-7.63	7.01E-13	1.86E-12
3-Phenyllactic acid	1.42	7.55	1.12E-12	2.93E-12
Aspartic acid	1.27	7.47	1.87E-12	4.82E-12
Asparagine	1.23	6.98	3.44E-11	8.73E-11
Gluconic acid	1.44	6.68	1.90E-10	4.75E-10
Phenylacetic acid	1.09	6.60	3.03E-10	7.47E-10
N-Acetylserine	1.29	6.57	3.46E-10	8.41E-10
Glycerol	1.19	6.54	4.18E-10	1.00E-09
Dihydroxyacetone phosphate	1.36	6.41	8.91E-10	2.10E-09
Mannitol	1.11	5.95	1.04E-08	2.42E-08
Fumaric acid	1.15	5.90	1.32E-08	3.03E-08
Ascorbic acid	1.16	5.90	1.35E-08	3.06E-08
Xanthine	1.27	5.85	1.75E-08	3.92E-08
Quinolinic acid	1.28	5.83	1.92E-08	4.24E-08
Histidine	1.22	5.82	2.09E-08	4.55E-08
Hippuric acid	1.11	5.59	6.68E-08	1.44E-07
Hypoxanthine	1.36	5.58	6.94E-08	1.47E-07
Isovalerylglycine	1.25	5.57	7.50E-08	1.57E-07
Aconitic acid	1.31	5.56	7.64E-08	1.58E-07
Fructose	1.10	5.41	1.62E-07	3.32E-07
Eicosapentaenoic acid	1.15	5.27	3.23E-07	6.55E-07
N-Acetyl-Lysine	1.11	5.20	4.59E-07	9.19E-07
3-Aminoglutaric acid	1.18	5.14	5.95E-07	1.18E-06
Lactic acid	1.18	5.10	7.32E-07	1.43E-06
Glucosamine	1.10	4.83	2.59E-06	5.01E-06
Ureidosuccinic acid	1.13	4.81	2.82E-06	5.39E-06
Glyceric acid	1.10	4.73	4.04E-06	7.64E-06
5-Dehydroquinic acid	1.12	4.62	6.61E-06	1.23E-05
Pyridoxine	1.12	4.41	1.63E-05	3.02E-05
Pyridoxal	1.10	4.34	2.17E-05	3.97E-05

Xylulose	1.12	4.22	3.50E-05	6.33E-05
2-Keto-isovaleric acid	1.10	4.13	5.05E-05	9.03E-05
Decanoic acid	1.10	4.03	7.81E-05	1.38E-04
Galacturonic acid	1.10	3.82	1.71E-04	2.99E-04
Margaric acid	1.10	3.70	2.69E-04	4.67E-04
Glucaric acid	0.87	-3.68	2.98E-04	5.12E-04
Mannose	1.05	3.65	3.29E-04	5.60E-04
2-Hydroxybutyric acid	1.14	3.49	5.75E-04	9.67E-04
Ribulose	1.09	3.46	6.59E-04	1.10E-03
Monostearin	1.07	3.44	6.97E-04	1.15E-03
N-Acetylneuraminic acid	2.69	3.38	8.68E-04	1.42E-03
Galactose	1.05	3.36	9.27E-04	1.50E-03
Sucrose	2.71	3.35	9.47E-04	1.52E-03
Glucose	1.05	3.33	1.00E-03	1.59E-03
ParaXanthine	1.08	3.31	1.10E-03	1.72E-03
Erythrulose	1.06	3.27	1.23E-03	1.92E-03
Glucono-1,5-lactone	1.05	3.24	1.38E-03	2.13E-03
Uracil	1.16	3.06	2.47E-03	3.78E-03
Azelaic acid	1.50	3.02	2.82E-03	4.29E-03
Arabitol	1.93	2.95	3.50E-03	5.26E-03
Citric acid	1.06	2.95	3.53E-03	5.27E-03
Ribitol	1.78	2.81	5.41E-03	8.00E-03
Pantothenic acid	1.41	2.74	6.60E-03	9.67E-03
Glucuronic acid	1.05	2.74	6.66E-03	9.68E-03
2-Aminopimelic acid	1.05	2.73	6.78E-03	9.77E-03
Rhamnose	2.71	2.73	6.94E-03	9.91E-03
Dihydrouracil	1.16	2.72	7.06E-03	1.00E-02
Sorbose	1.51	2.69	7.75E-03	1.09E-02
Lauric acid	1.09	2.66	8.33E-03	1.16E-02
Inositol	1.03	2.63	9.11E-03	1.26E-02
Psicose	1.51	2.60	1.00E-02	1.37E-02
N-Butyrylglycine	1.06	2.58	1.06E-02	1.44E-02
Tagatose	1.52	2.57	1.08E-02	1.46E-02
Citramalic acid	1.13	2.54	1.16E-02	1.56E-02
3-Hydroxyanthranilic acid	1.07	2.53	1.22E-02	1.62E-02
Myristic acid	1.05	2.52	1.25E-02	1.65E-02
2-Aminoethanol	1.03	2.44	1.56E-02	2.04E-02
Glyoxylic acid-oxime	1.03	2.39	1.79E-02	2.32E-02
2-Aminoisobutyric acid	1.06	2.22	2.72E-02	3.50E-02
Xylitol	1.05	2.21	2.79E-02	3.56E-02
Threonic acid	0.89	-2.14	3.38E-02	4.29E-02

Table S6. Significantly changed metabolites from mid-late gestation to 1 month postpartum in the PPD group (FDR q-value < 0.05) (EPDS ≥ 9; n = 209), Related to Figure 4A. Fold change, postpartum/pregnancy.

Metabolite	Fold change	t.stat	p.value	FDR
Ornithine	2.99	33.78	2.19E-86	3.72E-84
Glycine	2.22	29.90	3.03E-77	2.57E-75
Glycyl-Glycine	3.45	22.59	7.00E-58	3.97E-56
Serine	1.73	22.09	1.91E-56	8.11E-55
Uric acid	1.53	21.87	7.98E-56	2.71E-54
Tyrosine	1.75	21.65	3.52E-55	9.95E-54
Pyridoxamine	1.79	21.62	4.10E-55	9.95E-54
Dopa	1.51	19.84	7.54E-50	1.60E-48
Proline	1.94	19.72	1.70E-49	3.21E-48
Maleic acid	2.26	18.25	4.78E-45	8.12E-44
1,5-Anhydro-glucitol	1.74	18.16	8.59E-45	1.33E-43
Methionine sulfone	3.00	17.87	6.79E-44	9.61E-43
Acetylglycine	2.09	16.75	1.92E-40	2.51E-39
Dihydroxyacetone	1.49	16.23	8.18E-39	9.93E-38
Phenylalanine	1.40	15.89	9.34E-38	1.06E-36
Arginine	1.91	14.82	2.17E-34	2.31E-33
Creatinine	1.94	14.58	1.20E-33	1.20E-32
4-Hydroxyphenyllactic acid	1.74	14.45	3.25E-33	3.07E-32
Citrulline	2.11	14.33	7.56E-33	6.76E-32
Kynurenine	1.58	13.92	1.49E-31	1.27E-30
Methylmalonic acid	1.45	13.80	3.47E-31	2.81E-30
Valine	1.40	13.71	6.49E-31	5.02E-30
Glutamic acid	1.48	13.47	3.77E-30	2.79E-29
Glyceraldehyde	1.40	13.35	8.78E-30	6.22E-29
4-Hydroxyproline	1.60	12.22	3.05E-26	2.08E-25
Tyramine	1.33	11.86	4.01E-25	2.62E-24
Lysine	1.33	11.78	7.09E-25	4.46E-24
Isoleucine	1.45	11.75	9.07E-25	5.51E-24
Alanine	1.34	11.66	1.67E-24	9.76E-24
Leucine	1.40	11.59	2.68E-24	1.52E-23
Tryptophan	1.29	11.32	1.80E-23	9.88E-23
5-Oxoproline	1.50	11.30	2.22E-23	1.18E-22
Hypotaurine	1.65	11.17	5.44E-23	2.80E-22
2-Ketoglutaric acid	1.33	11.04	1.30E-22	6.49E-22
Urea	1.24	10.97	2.20E-22	1.07E-21
Glycolic acid	1.19	10.93	2.84E-22	1.34E-21
Isocitric acid	1.33	10.77	8.90E-22	4.09E-21
Succinic acid	1.32	10.38	1.37E-20	6.12E-20
Cysteine	1.35	10.24	3.38E-20	1.47E-19
Cystine	1.53	10.00	1.75E-19	7.44E-19
N-Acetylglutamine	1.37	9.96	2.37E-19	9.81E-19
Glutaric acid	1.92	9.75	1.00E-18	4.06E-18
2-Hydroxyisobutyric acid	1.38	9.72	1.16E-18	4.57E-18
Phosphoric acid	1.16	9.62	2.32E-18	8.97E-18

Indol-3-acetic acid	2.12	9.22	3.39E-17	1.28E-16
Tryptamine	2.31	9.00	1.40E-16	5.19E-16
3-Methyl-2-oxovaleric acid	1.19	8.89	2.94E-16	1.07E-15
Methionine	1.34	8.84	4.04E-16	1.43E-15
Fumaric acid	1.23	8.66	1.33E-15	4.63E-15
2-Ketoisocaproic acid	1.21	8.46	4.81E-15	1.64E-14
Glycerol	1.27	8.26	1.69E-14	5.63E-14
N-Acetylserine	1.41	7.80	2.88E-13	9.43E-13
2-Deoxy-glucose	1.34	7.77	3.56E-13	1.14E-12
Glutamine	1.33	7.64	7.60E-13	2.39E-12
2-Hydroxyglutaric acid	1.16	7.43	2.79E-12	8.62E-12
3-Phenyllactic acid	1.39	7.42	2.98E-12	9.03E-12
Malic acid	1.31	7.35	4.34E-12	1.29E-11
Dihydroorotic acid	1.34	7.14	1.50E-11	4.40E-11
Ribonic acid	1.24	6.72	1.72E-10	4.96E-10
Dihydroxyacetone phosphate	1.42	6.54	4.67E-10	1.32E-09
2-Aminoadipic acid	1.32	6.46	7.35E-10	2.05E-09
2-Hydroxyisovaleric acid	1.14	6.30	1.77E-09	4.85E-09
Ascorbic acid	1.17	6.28	1.95E-09	5.26E-09
Hypoxanthine	1.53	6.27	2.08E-09	5.53E-09
Quinolinic acid	1.41	6.10	5.20E-09	1.36E-08
Isovalerylglycine	1.34	6.01	8.05E-09	2.07E-08
3-Aminopropanoic acid	1.25	5.95	1.14E-08	2.89E-08
Aspartic acid	1.18	5.69	4.26E-08	1.07E-07
Uracil	1.41	5.66	4.92E-08	1.21E-07
Ethylmalonic acid	1.24	5.49	1.15E-07	2.79E-07
Homocysteine	1.49	5.41	1.70E-07	4.07E-07
Pantothenic acid	1.53	5.34	2.39E-07	5.65E-07
Gluconic acid	1.35	5.26	3.59E-07	8.36E-07
Aconitic acid	1.27	5.16	5.69E-07	1.31E-06
Threonine	0.87	-5.05	9.69E-07	2.20E-06
Asparagine	1.16	4.84	2.53E-06	5.67E-06
Xanthine	1.27	4.48	1.25E-05	2.76E-05
2-Hydroxybutyric acid	1.18	4.46	1.34E-05	2.93E-05
Glucaric acid	0.81	-4.28	2.83E-05	6.08E-05
Decanoic acid	1.12	4.21	3.79E-05	8.06E-05
Lauric acid	1.23	4.13	5.26E-05	1.10E-04
3-Aminoglutaric acid	1.12	4.00	8.68E-05	1.80E-04
Xylulose	1.11	3.97	1.00E-04	2.04E-04
Mannitol	1.10	3.96	1.01E-04	2.04E-04
2-Keto-isovaleric acid	1.09	3.92	1.22E-04	2.43E-04
Lactic acid	1.13	3.89	1.36E-04	2.69E-04
Arabitol	1.96	3.88	1.43E-04	2.79E-04
3-Sulfinoalanine	1.39	3.87	1.48E-04	2.86E-04
Cytosine	1.17	3.85	1.56E-04	2.98E-04
N-Acetyl-Lysine	1.09	3.84	1.65E-04	3.13E-04
Ureidosuccinic acid	1.11	3.82	1.77E-04	3.31E-04
Phenylpyruvic acid	0.78	-3.80	1.87E-04	3.46E-04
Ribitol	1.86	3.78	2.04E-04	3.72E-04
Pyridoxine	1.11	3.77	2.12E-04	3.83E-04
Rhamnose	2.59	3.75	2.25E-04	4.02E-04

Fructose	1.08	3.75	2.27E-04	4.02E-04
Hippuric acid	1.08	3.74	2.42E-04	4.24E-04
5-Dehydroquinic acid	1.09	3.65	3.32E-04	5.76E-04
ParaXanthine	1.13	3.64	3.39E-04	5.81E-04
Inositol	1.06	3.62	3.75E-04	6.38E-04
Glucosamine	1.08	3.53	5.20E-04	8.76E-04
Xylose	1.77	3.43	7.21E-04	1.20E-03
Ribulose	1.11	3.33	1.02E-03	1.68E-03
Pyridoxal	1.08	3.33	1.03E-03	1.68E-03
Glyceric acid	1.07	3.27	1.28E-03	2.07E-03
Palmitoleic acid	1.14	3.25	1.36E-03	2.18E-03
Dihydrouracil	1.15	3.20	1.62E-03	2.57E-03
Histidine	1.11	3.13	1.99E-03	3.12E-03
Malonic acid	1.17	3.09	2.29E-03	3.57E-03
Galacturonic acid	1.07	3.08	2.34E-03	3.61E-03
Hydroxylamine	0.91	-3.07	2.43E-03	3.73E-03
Psicose	1.52	3.00	3.00E-03	4.55E-03
Eicosapentaenoic acid	1.06	2.98	3.23E-03	4.86E-03
Glycerol 3-phosphate	1.27	2.96	3.46E-03	5.16E-03
Sorbose	1.51	2.95	3.49E-03	5.16E-03
Arabinose	1.74	2.87	4.55E-03	6.67E-03
Tagatose	1.51	2.85	4.77E-03	6.89E-03
Erythrulose	0.95	-2.85	4.79E-03	6.89E-03
N-Acetylmannosamine	1.04	2.73	6.95E-03	9.93E-03
Lyxose	1.76	2.70	7.51E-03	1.06E-02
Pyruvic acid	0.89	-2.51	1.30E-02	1.82E-02
Linoleic acid	1.10	2.44	1.55E-02	2.16E-02
2-Propyl-5-hydroxy-pentanoic	0.94	-2.39	1.79E-02	2.48E-02
Myristic acid	1.04	2.35	1.95E-02	2.67E-02
Monostearin	0.97	-2.27	2.45E-02	3.33E-02
3-Hydroxyisovaleric acid	0.95	-2.22	2.78E-02	3.76E-02
Dimethylglycine	0.96	-2.20	2.87E-02	3.84E-02
Triethanolamine	1.23	2.19	2.96E-02	3.93E-02
Elaidic acid	1.09	2.16	3.17E-02	4.18E-02
Citric acid	1.05	2.15	3.30E-02	4.31E-02
Oleic acid	1.09	2.11	3.60E-02	4.67E-02

Table S7. Significantly enriched pathways of changed metabolites from mid-late gestation to 1 month postpartum in the control group (EPDS ≤ 2; n = 222), Related to Figure 4B. Holm-Bofferoni correction (Holm adjust) was used as a post hoc method in multiple comparisons to verify the specific difference in each.

Pathway	Total	Expected	Hits	Raw p	Holm adjust
Aminoacyl-tRNA biosynthesis	48	4.21	19	3.19E-09	2.68E-07
Arginine biosynthesis	14	1.23	10	1.46E-08	1.21E-06
Valine, leucine and isoleucine biosynthesis	8	0.70	7	2.57E-07	2.11E-05
Alanine, aspartate and glutamate metabolism	28	2.46	10	6.49E-05	5.25E-03
Pantothenate and CoA biosynthesis	19	1.67	8	9.45E-05	7.56E-03
Phenylalanine metabolism	10	0.88	5	8.50E-04	6.71E-02
Phenylalanine, tyrosine and tryptophan biosynthesis	4	0.35	3	2.48E-03	1.93E-01
Glyoxylate and dicarboxylate metabolism	32	2.81	8	4.90E-03	3.78E-01
Citrate cycle (TCA cycle)	20	1.75	6	5.64E-03	4.29E-01
Glycerolipid metabolism	16	1.40	5	9.55E-03	7.16E-01
D-Glutamine and D-glutamate metabolism	6	0.53	3	1.08E-02	8.03E-01
Ascorbate and aldarate metabolism	8	0.70	3	2.66E-02	1.00E+00
beta-Alanine metabolism	21	1.84	5	3.11E-02	1.00E+00
Butanoate metabolism	15	1.32	4	3.61E-02	1.00E+00
Vitamin B6 metabolism	9	0.79	3	3.74E-02	1.00E+00
Valine, leucine and isoleucine degradation	40	3.51	7	5.48E-02	1.00E+00
Glycine, serine and threonine metabolism	33	2.90	6	6.26E-02	1.00E+00
Pentose and glucuronate interconversions	18	1.58	4	6.59E-02	1.00E+00
Nitrogen metabolism	6	0.53	2	9.07E-02	1.00E+00
Glutathione metabolism	28	2.46	5	9.18E-02	1.00E+00
Arginine and proline metabolism	38	3.33	6	1.09E-01	1.00E+00
Pyrimidine metabolism	39	3.42	6	1.20E-01	1.00E+00
Taurine and hypotaurine metabolism	8	0.70	2	1.51E-01	1.00E+00
Cysteine and methionine metabolism	33	2.90	5	1.57E-01	1.00E+00
Histidine metabolism	16	1.40	3	1.60E-01	1.00E+00
Galactose metabolism	27	2.37	4	2.07E-01	1.00E+00

Tryptophan metabolism	41	3.60	5	2.88E-01	1.00E+00
Pentose phosphate pathway	22	1.93	3	3.03E-01	1.00E+00
Propanoate metabolism	23	2.02	3	3.28E-01	1.00E+00
Synthesis and degradation of ketone bodies	5	0.44	1	3.69E-01	1.00E+00
Linoleic acid metabolism	5	0.44	1	3.69E-01	1.00E+00
Nicotinate and nicotinamide metabolism	15	1.32	2	3.84E-01	1.00E+00
Biosynthesis of unsaturated fatty acids	36	3.16	4	3.90E-01	1.00E+00
Glycolysis / Gluconeogenesis	26	2.28	3	4.03E-01	1.00E+00
Thiamine metabolism	7	0.61	1	4.75E-01	1.00E+00
Inositol phosphate metabolism	30	2.63	3	4.98E-01	1.00E+00
Tyrosine metabolism	42	3.69	4	5.11E-01	1.00E+00
Purine metabolism	65	5.70	6	5.13E-01	1.00E+00
Ubiquinone and other terpenoid-quinone biosynthesis	9	0.79	1	5.63E-01	1.00E+00
Pyruvate metabolism	22	1.93	2	5.89E-01	1.00E+00
Biotin metabolism	10	0.88	1	6.02E-01	1.00E+00
Caffeine metabolism	10	0.88	1	6.02E-01	1.00E+00
Fatty acid biosynthesis	47	4.12	4	6.03E-01	1.00E+00
Glycerophospholipid metabolism	36	3.16	3	6.26E-01	1.00E+00
Amino sugar and nucleotide sugar metabolism	37	3.25	3	6.44E-01	1.00E+00
Lysine degradation	25	2.19	2	6.60E-01	1.00E+00
Porphyrin and chlorophyll metabolism	30	2.63	2	7.56E-01	1.00E+00
Starch and sucrose metabolism	18	1.58	1	8.10E-01	1.00E+00
Selenocompound metabolism	20	1.75	1	8.43E-01	1.00E+00
Fructose and mannose metabolism	20	1.75	1	8.43E-01	1.00E+00
Phosphatidylinositol signaling system	28	2.46	1	9.25E-01	1.00E+00
Fatty acid degradation	39	3.42	1	9.73E-01	1.00E+00
Primary bile acid biosynthesis	46	4.04	1	9.86E-01	1.00E+00

Table S8. Significantly enriched pathways of changed metabolites from mid-late gestation to 1 month postpartum in the control group (EPDS ≥ 9; n = 209), Related to Figure 4B. Holm–Bonferroni method (Holm adjust) was used as a post hoc method in multiple comparisons to verify the specific difference in each.

Pathway	Total	Expected	Hits	Raw p	Holm adjust
Aminoacyl-tRNA biosynthesis	48	3.87	19	7.04E-10	5.91E-08
Arginine biosynthesis	14	1.13	10	6.26E-09	5.19E-07
Valine, leucine and isoleucine biosynthesis	8	0.65	7	1.41E-07	1.16E-05
Alanine, aspartate and glutamate metabolism	28	2.26	11	4.01E-06	3.25E-04
Pantothenate and CoA biosynthesis	19	1.53	8	5.09E-05	4.07E-03
Citrate cycle (TCA cycle)	20	1.61	7	5.99E-04	4.73E-02
Phenylalanine, tyrosine and tryptophan biosynthesis	4	0.32	3	1.93E-03	1.51E-01
Glyoxylate and dicarboxylate metabolism	32	2.58	8	2.88E-03	2.22E-01
Phenylalanine metabolism	10	0.81	4	5.78E-03	4.39E-01
Glycerolipid metabolism	16	1.29	5	6.66E-03	5.00E-01
D-Glutamine and D-glutamate metabolism	6	0.48	3	8.55E-03	6.33E-01
Taurine and hypotaurine metabolism	8	0.65	3	2.12E-02	1.00E+00
beta-Alanine metabolism	21	1.69	5	2.24E-02	1.00E+00
Vitamin B6 metabolism	9	0.73	3	3.00E-02	1.00E+00
Valine, leucine and isoleucine degradation	40	3.23	7	3.70E-02	1.00E+00
Glycine, serine and threonine metabolism	33	2.66	6	4.42E-02	1.00E+00
Cysteine and methionine metabolism	33	2.66	6	4.42E-02	1.00E+00
Glutathione metabolism	28	2.26	5	6.88E-02	1.00E+00
Nitrogen metabolism	6	0.48	2	7.81E-02	1.00E+00
Arginine and proline metabolism	38	3.06	6	7.94E-02	1.00E+00
Pyrimidine metabolism	39	3.15	6	8.79E-02	1.00E+00
Butanoate metabolism	15	1.21	3	1.14E-01	1.00E+00
Ascorbate and aldarate metabolism	8	0.65	2	1.31E-01	1.00E+00
Histidine metabolism	16	1.29	3	1.33E-01	1.00E+00
Biosynthesis of unsaturated fatty acids	36	2.90	5	1.59E-01	1.00E+00
Tryptophan metabolism	41	3.31	5	2.31E-01	1.00E+00

Tyrosine metabolism	42	3.39	5	2.47E-01	1.00E+00
Pyruvate metabolism	22	1.77	3	2.59E-01	1.00E+00
Propanoate metabolism	23	1.85	3	2.82E-01	1.00E+00
Fatty acid biosynthesis	47	3.79	5	3.27E-01	1.00E+00
Linoleic acid metabolism	5	0.40	1	3.44E-01	1.00E+00
Nicotinate and nicotinamide metabolism	15	1.21	2	3.44E-01	1.00E+00
Glycolysis / Gluconeogenesis	26	2.10	3	3.51E-01	1.00E+00
Pentose and glucuronate interconversions	18	1.45	2	4.33E-01	1.00E+00
Thiamine metabolism	7	0.56	1	4.46E-01	1.00E+00
Ubiquinone and other terpenoid-quinone biosynthesis	9	0.73	1	5.32E-01	1.00E+00
Pentose phosphate pathway	22	1.77	2	5.41E-01	1.00E+00
Biotin metabolism	10	0.81	1	5.70E-01	1.00E+00
Caffeine metabolism	10	0.81	1	5.70E-01	1.00E+00
Amino sugar and nucleotide sugar metabolism	37	2.98	3	5.85E-01	1.00E+00
Lysine degradation	25	2.02	2	6.12E-01	1.00E+00
Purine metabolism	65	5.24	5	6.14E-01	1.00E+00
Galactose metabolism	27	2.18	2	6.55E-01	1.00E+00
Porphyrin and chlorophyll metabolism	30	2.42	2	7.11E-01	1.00E+00
Inositol phosphate metabolism	30	2.42	2	7.11E-01	1.00E+00
Glycerophospholipid metabolism	36	2.90	2	8.02E-01	1.00E+00
Selenocompound metabolism	20	1.61	1	8.16E-01	1.00E+00
Fructose and mannose metabolism	20	1.61	1	8.16E-01	1.00E+00
Fatty acid degradation	39	3.15	2	8.37E-01	1.00E+00
Primary bile acid biosynthesis	46	3.71	2	8.98E-01	1.00E+00
Phosphatidylinositol signaling system	28	2.26	1	9.07E-01	1.00E+00
Fatty acid elongation	39	3.15	1	9.64E-01	1.00E+00
Steroid biosynthesis	42	3.39	1	9.72E-01	1.00E+00
Steroid hormone biosynthesis	85	6.85	1	9.99E-01	1.00E+00

Table S9. List of differentially altered metabolites from pregnancy to postpartum between control (EPDS ≤ 2; n = 222) and postpartum depression (EPDS ≥ 9; n = 209) groups, Related to Figure 4C. PPD, postpartum depression. NA, not applicable.

Metabolite	HMDB ID	KEGG	Control postpartum/pregnancy (fold change)	Control postpartum/pregnancy (FDR)	Control Increased ↑ Decreased ↓	PPD postpartum/pregnancy (fold change)	PPD postpartum/pregnancy (FDR)	PPD Increased ↑ Decreased ↓
**2-Aminoethanol**	HMDB0000149	C00189	1.032	2.04E-02	↑	1.025	1.31E-01	
**2-Aminoisobutyric acid**	HMDB0001906	C03665	1.059	3.50E-02	↑	0.987	7.85E-01	
**2-Aminopimelic acid**	HMDB0034252	NA	1.050	9.77E-03	↑	1.047	5.24E-02	
**2-Propyl-5-hydroxy-pentanoic acid**	HMDB0013898	C16650	1.061	7.64E-02		0.938	2.48E-02	↓
**3-Hydroxyanthranilic acid**	HMDB0001476	C00632	1.068	1.62E-02	↑	1.094	5.15E-02	
**3-Hydroxyisovaleric acid**	HMDB0000754	C20827	1.029	3.20E-01		0.947	3.76E-02	↓
**3-Sulfinoalanine**	HMDB0000996	C00606	1.040	2.92E-01		1.386	2.86E-04	↑
**Azelaic acid**	HMDB0000784	C08261	1.504	4.29E-03	↑	1.069	8.54E-02	
**Citramalic acid**	HMDB0000426	C00815	1.134	1.56E-02	↑	1.057	5.91E-02	
**Cytosine**	HMDB0000630	C00380	0.933	1.20E-01		1.169	2.98E-04	↑
**Dimethylglycine**	HMDB0000092	C01026	0.966	1.82E-01		0.959	3.84E-02	↓
**Elaidic acid**	HMDB0000573	C01712	0.996	5.45E-02		1.095	4.18E-02	↑
**Erythrulose**	HMDB0006293	C02045	1.055	1.92E-03	↑	0.954	6.89E-03	↓
**Galactose**	HMDB0000143	C00984	1.047	1.50E-03	↑	0.995	8.58E-01	
**Glucono-1,5-lactone**	HMDB0000150	C00198	1.048	2.13E-03	↑	0.996	9.02E-01	
**Glucose**	HMDB0000122	C00221	1.048	1.59E-03	↑	0.995	8.21E-01	
**Glucuronic acid**	HMDB0000127	C00191	1.055	9.68E-03	↑	0.995	9.39E-01	
**Glycerol 3-phosphate**	HMDB0000126	C00093	1.130	8.42E-02		1.266	5.16E-03	↑
**Glyoxylic acid-oxime**	HMDB0000119	C00048	1.033	2.32E-02	↑	0.976	1.04E-01	
**Hydroxylamine**	HMDB0003338	C00192	1.030	4.75E-01		0.908	3.73E-03	↓
**Linoleic acid**	HMDB0000673	C01595	1.036	5.60E-02		1.098	2.16E-02	↑
**Malonic acid**	HMDB0000691	C04025	1.071	6.10E-02		1.168	3.57E-03	↑
**Mannose**	HMDB0000169	C00936	1.049	5.60E-04	↑	0.999	9.98E-01	
**Margaric acid**	HMDB0002259	NA	1.099	4.67E-04	↑	0.995	9.68E-01	
**Monostearin**	HMDB0031075	NA	1.066	1.15E-03	↑	0.966	3.33E-02	↓
**N-Acetylmannosamine**	HMDB0001129	C00645	1.011	7.58E-01		1.043	9.93E-03	↑
**N-Acetylneuraminic acid**	HMDB0000230	C19910	2.692	1.42E-03	↑	2.430	7.89E-01	
**N-Butyrylglycine**	HMDB0000808	NA	1.058	1.44E-02	↑	0.983	4.32E-01	

**Oleic acid**	HMDB0000207	C00712	0.997	5.39E-02		1.091	4.67E-02	↑
**Palmitoleic acid**	HMDB0003229	C08362	0.927	5.70E-02		1.144	2.18E-03	↑
**Phenylacetic acid**	HMDB0000209	C07086	1.094	7.47E-10	↑	1.013	4.85E-01	
**Phenylpyruvic acid**	HMDB0000205	C00166	0.904	7.17E-02		0.779	3.46E-04	↓
**Pyruvic acid**	HMDB0000243	C00022	1.001	3.44E-01		0.888	1.82E-02	↓
**Sucrose**	HMDB0000258	C00089	2.707	1.52E-03	↑	2.401	7.89E-01	
**Threonic acid**	HMDB0000943	C01620	0.888	4.29E-02	↓	0.895	8.80E-02	
**Triethanolamine**	HMDB0032538	C06771	1.225	3.45E-01		1.229	3.93E-02	↑
**Xylitol**	HMDB0002917	C00379	1.049	3.56E-02	↑	0.990	6.53E-01	
